# Data cleaning and management protocols for linked perinatal research data: a good practice example from the Smoking MUMS (Maternal Use of Medications and Safety) Study

**DOI:** 10.1186/s12874-017-0385-6

**Published:** 2017-07-11

**Authors:** Duong Thuy Tran, Alys Havard, Louisa R. Jorm

**Affiliations:** Centre for Big Data Research in Health, Faculty of Medicine, UNSW Sydney (The University of New South Wales), Sydney, NSW 2052 Australia

**Keywords:** Data cleaning methods, Data consistency, Perinatal, Record linkage

## Abstract

**Background:**

Data cleaning is an important quality assurance in data linkage research studies. This paper presents the data cleaning and preparation process for a large-scale cross-jurisdictional Australian study (the Smoking MUMS Study) to evaluate the utilisation and safety of smoking cessation pharmacotherapies during pregnancy.

**Methods:**

Perinatal records for all deliveries (2003–2012) in the States of New South Wales (NSW) and Western Australia were linked to State-based data collections including hospital separation, emergency department and death data (mothers and babies) and congenital defect notifications (babies in NSW) by State-based data linkage units. A national data linkage unit linked pharmaceutical dispensing data for the mothers. All linkages were probabilistic. Twenty two steps assessed the uniqueness of records and consistency of items within and across data sources, resolved discrepancies in the linkages between units, and identified women having records in both States.

**Results:**

State-based linkages yielded a cohort of 783,471 mothers and 1,232,440 babies. Likely false positive links relating to 3703 mothers were identified. Corrections of baby’s date of birth and age, and parity were made for 43,578 records while 1996 records were flagged as duplicates. Checks for the uniqueness of the matches between State and national linkages detected 3404 ID clusters, suggestive of missed links in the State linkages, and identified 1986 women who had records in both States.

**Conclusions:**

Analysis of content data can identify inaccurate links that cannot be detected by data linkage units that have access to personal identifiers only. Perinatal researchers are encouraged to adopt the methods presented to ensure quality and consistency among studies using linked administrative data.

**Electronic supplementary material:**

The online version of this article (doi:10.1186/s12874-017-0385-6) contains supplementary material, which is available to authorized users.

## Background

The linkage of routinely collected perinatal and other administrative health data has broadened the scope of maternal and child health research as it enables researchers to establish and follow-up large samples or whole populations and ascertain multiple factors for risk adjustment [1]. Linking perinatal to pharmaceutical dispensing data offers a valuable approach for pharmacovigilance and examination of medication safety in pregnancy [2] given ethical concerns about including pregnant women in clinical trials [3] and bias associated with voluntary reporting to post-market pharmaceutical surveillance systems [4].

In many countries, including Australia, unique individual identifiers are not available across all of the administrative data collections relevant to perinatal research. In this situation, probabilistic linkage methods are used to link individuals’ records [5, 6], but probabilistic linkage is not perfect [7]. Previous studies have reported that the sensitivity (i.e. truly matched records) of probabilistic linkage ranges from 74 to 98%, and specificity (i.e. truly unmatched records) ranges between 99 and 100% [8]. False and missed matches can introduce bias and affect the validity of research findings. While data linkage units aim to improve the quality of linkage, there is a growing consensus that data cleaning (i.e. detecting, diagnosing, and editing data anomalies) [9] and proper documentation are essential aspects of quality assurance [9–11]. The RECORD Statement recommends that observational studies using routinely collected health data should provide information on the process and quality of linkage and data cleaning [10]. Furthermore, systematic checks have a potential to improve quality of future linkage through provision of feedback to data linkage units.

In studies that involve cross-jurisdictional linkages, additional data cleaning considerations are required. Australia has a federated health care system with delivery and administration of services being the responsibility of either States/Territories (e.g. hospital services) or the Federal government (e.g. subsidised pharmaceuticals). In this setting, cross-jurisdictional linkage brings together diverse and rich data sources, enabling national-level research studies [12]. Cross-jurisdictional linkage performed by different data linkage units, however, is subject to discrepancies resulting from variations in the use of personal identifiers, techniques for constructing linkage keys and quality assurance policies. Consistency checks, therefore, are vital before merging records from different States.

Cleaning linked data is a complex process and requires thorough planning and knowledge about data collection methodologies and the validity of the data items. While there are existing frameworks and check lists for data cleaning [9, 11], literature that describes how to systematically examine the consistency of content data in linked perinatal records [13], and how to identify and resolve disparities arising from cross-jurisdictional linkages is lacking. Additionally, researchers rarely provide their coding syntax, making it difficult to replicate their data cleaning procedures. This paper presents a series of steps for assessing data consistency and cleaning in the Smoking MUMS (Maternal Use of Medications and Safety) Study [14] which involves the linkage of perinatal records from two Australian states—New South Wales (NSW) and Western Australia (WA)—to national Pharmaceutical Benefits Scheme (PBS) claims data. Exemplar documentation and SAS code presented in the paper can be adopted in similar studies.

## Methods

### Study design and data sources

**Fig. 1:**
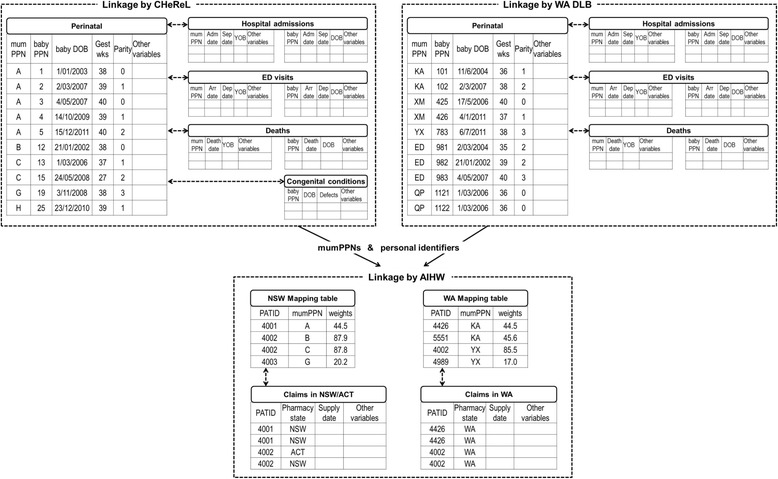
Data linkage and examples of data set layouts

The Smoking MUMS Study is an observational cohort study including all women who delivered in NSW and WA between 1 January 2003 and 31 December 2012, and their babies. For mothers, perinatal records (i.e. the mother’s deliveries, including pre-2003 records) were linked to hospital separations (i.e. hospital discharge), emergency department (ED) attendances, death, and pharmaceutical claims records. For babies, perinatal records (i.e. the baby’s birth) were linked to hospital, ED and death data. Congenital defect notifications were included in the linkage for babies born in NSW (Fig. 1). New South Wales is Australia’s most populous State with more than 7.5 million residents, while WA has a population of 2.6 million [15]. Table 1 describes the data collections used in the study.

**Table 1:** Descriptions of data sets

Data type	Jurisdiction	Description	Number of records and persons prior to cleaning	Dates covered
Perinatal^a^				
Perinatal Data Collection (PDC)	NSW	Notifications of all livebirths and stillbirths of at least 20 weeks of gestation or at least 400 g birthweight in NSW and WA.	N records: 1155,207^b^ N persons: 595,457 mothers 937,345 babies	1/1/2003–31/12/2012^b^
Midwives Notification System (MNS)	WA		N records: 371155^b^ N persons: 188,014 mothers 295,095 babies	1/1/2003–31/12/2012^b^
Hospital admission^a^				
Admitted Patient Data Collection (APDC)	NSW	All hospital discharges from all public and private hospitals in NSW and WA.	Mother: 2,708,607 Baby: 1,835,852	Mother: 1/7/2001–30/6/2014 Baby: 1/1/2003–30/6/2014
Hospital Morbidity Data Collection (HMDC)	WA		Mother: 1,245,018 Baby: 307,620	Mother: 1/1/1980–30/6/2013 Baby: 1/1/2003–30/6/2013
Emergency department attendance				
Emergency Department Data Collection (EDDC)	NSW	ED attendances at all EDs in metropolitan areas and majority of EDs in regional areas in NSW and WA.	Mother: 1,518,745 Baby: 2,564,429	1/1/2005–31/12/2014
Emergency Department Data Collection (EDDC)	WA		Mother: 854,050 Baby: 1,017,206	Mother: 1/1/2002–30/11/2013 Baby: 1/1/2003–30/11/2013
Death data				
NSW Registry of Births, Deaths and Marriages (RBDM)	NSW	Date of deaths registered in NSW	Mother: 1337 Baby: 4271	1/1/2003–31/12/2014
Causes Of Death Unit Record File (COD URF)	NSW	Underlying and contributing causes of death for those registered in NSW	Mother: 881 Baby: 3897	1/1/2003–31/12/2012^c^
WA death registration and COD URF	WA	Date of death, underlying and contributing causes of death for those registered in WA	Mother: 478 Baby: 3345	1/1/2003–31/12/2013
Congenital Conditions				
Register of Congenital Conditions (RoCC)	NSW	Congenital conditions detected during pregnancy or at birth, or diagnosed in infants up to 1 year of age	Baby: 9976	1/1/2004–31/12/2009
PBS links mapping tables and claims data				
Mapping table between PBS PATIDs and NSW mumPPNs	Federal	All identified matches wherein the weight of the match ≥ 17 ^d^	1,006,960 matches	
Mapping table between PBS PATIDs and WA mumPPNs	Federal		226,817 matches	
PBS claim records, NSW or Australian Capital Territory (ACT)^e^ is the State of the pharmacies	Federal	Claims made in NSW and ACT for all PATIDs included in the NSW mapping table^e^	17,470,068 claim records	1/1/2003–31/12/2013
PBS claim records, WA is the State of the pharmacies	Federal	Claims made in WA for all PATIDs included in the WA mapping table^e^	3,364,490 claim records	1/1/2003–31/12/2013

^a^A child birth generates one birth notification (≥2 notifications if plural births), one hospital separation record for the mother, and one hospital separation record for the newborn (≥2 hospital records for the newborns if plural births)

^b^Records of pre-2003 deliveries (from 1994 in NSW, 1980 in WA) were included in the linkage for the mothers

^c^The NSW COD URF data are not available for the same duration as the RBDM death registrations due to the time needed for coding causes of death

^d^The recommended threshold weight to accept the matches to NSW mumPPNs and WA mumPPNs was 29 and 28, respectively

^e^The Australian Capital Territory (population 385,996) is geographically surrounded by NSW. PBS claims data for NSW women included those dispensed in NSW and the ACT and covered a wider range of pharmaceutical items than claims data for WA mothers

### Data linkage

All the linkages for the Smoking MUMS Study used probabilistic linkage methods and a privacy preserving approach [16–18]. Specifically, personal identifiers were separated from health information, with the data linkage units receiving personal identifiers only (i.e. no health information) and encrypted record IDs from the data custodians. The linkage units assigned a project-specific person number to all records that belonged to the same person and returned these person numbers and encrypted record IDs to the respective data custodians who released the approved research variables together with the person numbers (i.e. no personal identifiers) to the researchers [16–18].

In NSW, the Centre for Health Record Linkage (CHeReL) has established a Master Linkage Key to routinely link the Perinatal Data Collection with the other NSW data collections (Table 1), except the Register of Congenital Conditions which was specifically linked for NSW babies in this project. Likewise, the WA Data Linkage Branch (WA DLB) regularly links the Midwifery Notification System to the other WA data collections (Table 1). The Master Linkage Keys in NSW and WA are regularly updated and assessed via robust quality assurance procedures. The false positive rates for NSW and WA were estimated to be 0.3 and 0.05% respectively [19, 20]. Once the linkages for mother and baby cohorts were finalised, the CHeReL and WA DLB created a Project Person Number for each mother (mumPPN) and each baby (babyPPN, mapped to mumPPN).

In Australia, records of claims for pharmaceutical dispensing processed by the Federal government PBS are not routinely linked to State-based health records. For this study, PBS data custodian assigned a project-specific Patient Identification Number (PATID) to each woman who had claim records and provided PATIDs and personal details to the Australian Institute of Health and Welfare (AIHW) Integration Services Centre, while CHeReL and WA DLB provided the list of mumPPNs and identifiers (Fig. 1). The AIHW conducted probabilistic linkages based on personal identifiers and assigned weights (i.e. degree of similarity between the pairs of records, higher weights indicating greater similarity) to matches between PATIDs and PPNs. Based on AIHW clerical reviews, recommended threshold for accepting the matches to NSW mumPPNs was 29.0 (link rate 99.43%, link accuracy 98.62%) and 28.0 for matches to WA mumPPNs (link rate 99.02%, link accuracy 98.65%) [21]. Separate mapping tables for each State, including any PATID-PPN matches with weight ≥ 17 were released to researchers, as were separate files containing claims records relating to PATIDs that were included in the mapping tables (Table 1, Fig. 1). The release of claims records for matches with weights lower than the recommended threshold allows for sensitivity analyses in which different thresholds are used.

#### Steps to check consistency of State-based data

Prior to the assessment of data consistency, all data sets were examined to make sure that all variables and associated data dictionaries were delivered as expected, and the number of persons and records were in accordance with reports provided by the data linkage units. The mother’s hospital separation record and the child’s hospital separation record that correspond to the delivery of the mother and the birth of the child were carefully identified based on previously reported methods [6]. The range of data values, distribution by year and missing values were explored for all variables. Data items that underwent historical changes (as per data dictionaries or the published midwife notification forms) were examined whether the distribution of data is consistent with the documented changes (results not shown).

**Fig. 2:**
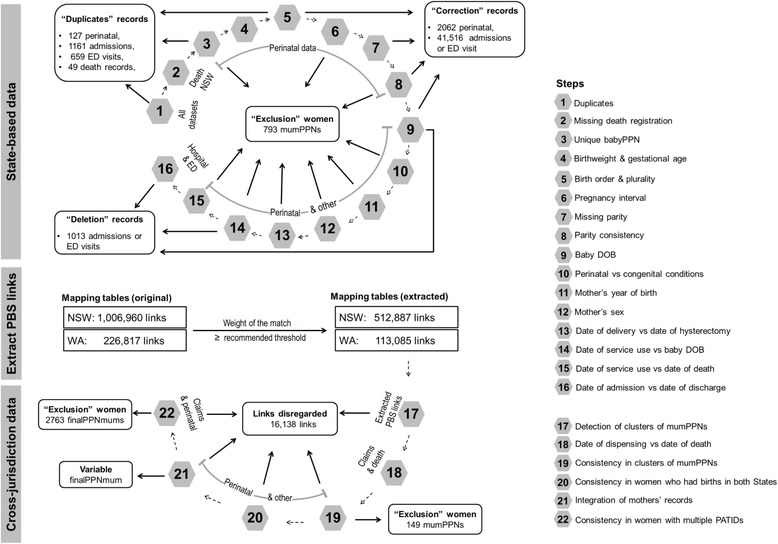
Summary of data cleaning steps and results

Consistency of State-based data was assessed through a series of steps (Fig. 2 and Table 2).

- Steps 1 to 3 examined the uniqueness of records.
- Steps 4 to 8 checked the consistency within and across pregnancies based on perinatal data items, including baby date of birth (DOB), parity, pregnancy plurality, birth order, gestational age, and birthweight. These variables were used because previous validation studies have reported high levels of accuracy in their recording [22, 23]. Parity was defined as the number of previous pregnancies ≥20 weeks and numerically coded (e.g. 0, 1, 2, 3). Plurality assigned pregnancies as single or multiple-fetus (coded as singleton, twins, triplets, quadruplets, etc.) while birth order indicated the order each baby was born (coded as 1st, 2nd, 3rd, etc.). Plural pregnancies generated more than one perinatal record which contained the same maternal information but baby-specific information, including order of birth. Gestational age was defined as number of completed weeks of gestation. Date of conception was calculated (baby DOB – completed weeks of gestation × 7 + 14 days).
- Steps 9 to 16 assessed the consistency of information across data sources, including consistency between unique events (birth, death) and episodes of health service use. These steps capitalised on the availability of the same information (e.g. baby DOB, interchangeably date of delivery, mother’s month and year of birth) in multiple data sets and validity of these variables [22, 23].

**Table 2:** Steps undertaken to assess consistency of State-based data

Step Data sets		Explanation^a^	Findings
Uniqueness of record			
1	Record duplicates		
	Perinatal, Hospital, ED, Death	1.1 Identify identical duplicates i.e. all variables contain the same information, except the unique ID of the records; 1.2 Identify partial duplicates for the same death: Remove identical duplicates (identified in Step 1.1). Among the remaining records, identify PPNs that present in ≥2 records, then review records of these PPNs, using information from other data sets if necessary^b^.	• Marked the records as “duplicate”: 9 perinatal, 1161 hospital admissions, 659 ED visits, and 49 death records.
2	Missing record of death registration (NSW only)		
	Death	2.1 Identify whether a PPN is absent from the death registration but present in the causes of death data set	• No cases found (as expected on the basis of deterministic linkage methodology for death data).
3	Uniqueness of babyPPN		
	Perinatal	Non-unique babyPPN may be due to linkage errors or multiple data entries; 3.1 Remove records flagged as “duplicate” (Step 1); 3.2 Identify babyPPNs that present in ≥2 perinatal records, then identify related mumPPNs; 3.3 Examine whether different mumPPNs are mapped to the same babyPPN. If this is the case, flag mothers as “exclusion”. 3.4 For the remaining mothers, review all of their perinatal records ^c^. Flag the mother as “exclusion” if the review suggests linkage error, otherwise mark the record as “duplicate”.	• 31 mothers were flagged as “exclusion” • 114 records were marked as “duplicate”.
Consistency of perinatal information			
4	Birthweight and gestational age		
	Perinatal	4.1 Cross-tabulate birthweight (categorised as missing, <400, 400–999, 1000–1999, 2000–4999, and ≥5000 g) with gestational age (categorised as missing, <20, 20–26, 27–36, 37–44, and ≥45 weeks); 4.2 Quantify unexpected records where gestation <20 weeks and birthweight <400g^d^; 4.3 Quantify outlier gestational age (≥45 weeks); 4.4 Quantify extreme birthweight (relative to gestational age); 4.5 No changes were made as outlier values could relate to medical reasons.	• 28 records with both gestation <20 weeks and birthweight <400 g; • 79 records with gestation ≥45 weeks; • 57 records with birthweight <1000 g and gestation ≥45 weeks; • 40 records with birthweight ≥2000 g and gestation ≤26 weeks.
5	Birth order and pregnancy plurality		
	Perinatal	It is necessary to select one record per delivery (where birth order = 1st) when pregnancy is the unit of analysis^e^. 5.1 Identify records with implausible order of birth given plurality (e.g. a singleton with birth order = 2nd, twins with birth order = 3rd); 5.2 Subset to plural pregnancies and sort according to baby DOB and ascending birth order. For each plural pregnancy, create the expected sequence of birth: sequence =1 for the first birth and increases by 1 for each subsequent birth, allowing for possibility that twins, triplets born on different dates^f^; 5.3 If the expected sequence of birth differs from the recorded birth order, then identify the mothers and review all of their perinatal records. Make correction if the review suggests typo error, otherwise mark the record as “duplicate”.	• No record with implausible birth order (given the plurality); • Correction made for 10 twins records (birth order changed from 1st to 2nd) and another 6 records (baby DOB); • 4 records marked as “duplicate”.
6	Interval between the two consecutive pregnancies		
	Perinatal	6.1 Select records where birth order = 1st and sort in ascending order of baby DOB; 6.2 Calculate date of conception (baby DOB – gestation weeks × 7 + 14 days); 6.3 Calculate the interval between two consecutive pregnancies (date of conception – date of prior delivery – 7 days), allowing for gestation being recorded as completed weeks; 6.4 Flag mother as “exclusion” if pregnancy interval < 0.	• 396 mothers marked as “exclusion”.
7	Missing value of parity		
	Perinatal	7.1 Subset to mothers who had a missing value of parity. 7.2 If the missing parity in a record relating to plural births, replace the missing with the value available in the other twins or triplets records. 7.3 After replacing missing parity in plural births, further subset to records where birth order = 1st. Then, for each mother: 7.3.1 Quantify how many pregnancies are recorded in the data (regardless of parity); 7.3.2 Quantify how many records with missing parity; 7.3.3 Examine whether parity in the second record is zero^g^; 7.3.4 Among records with a parity (non-missing), sort in ascending baby DOB, then categorise the sequence of parity as logical or illogical. The sequence is logical if between any two consecutive records, the parity value in the prior record is less than the value in the next record (e.g. parity values as 0-1-2-4); otherwise the sequence is illogical (e.g. parity values as 0-2-1); 7.4 Make no changes for mothers who either had only one pregnancy recorded, ≥2 records with missing data, parity as zero in the second record, or illogical parity sequence (further examine in Step 8); 7.5 Among the remaining mothers: Replace missing parity in the 7.5.1 first record (=next parity −1) if next parity equal to 1; or next parity > 1 and pregnancy interval < 40 weeks^h^; otherwise make no changes; 7.5.2 last record (=prior parity +1) if pregnancy interval < 40weeks^d^; 7.5.3 record other than the first and last (=prior parity +1) if the difference between the two adjacent parity values equal to 2; or the difference > 2 and interval < 40weeks^i^; otherwise make no changes.	• Missing parity was replaced for 1218 out of 1633 records.
8	Consistency in parity		
	Perinatal	8.1 Select records where birth order = 1st, sort in ascending baby DOB, then for each mother: 8.1.1 Count how many pregnancies are recorded in the data (regardless of parity); 8.1.2 Calculate the expected number of pregnancies (=highest parity value - lowest parity value +1); 8.1.3 Categorise the sequence of parity as logical or illogical (as per Step 7.3.4); 8.2 Among mothers who have logical sequence of parity: 8.2.1 Expected number of pregnancies equal to the count indicates parity consistency; 8.2.2 Expected number less than the count is due to missing data in parity (not replaced in Step 7). Make no changes; 8.2.3 Expected number greater than the count (by 1 to 3) suggests mother might have intervening births interstate or overseas (e.g. parity as 1-2-4-5); 8.2.4 Expected number substantially greater than count, especially the expected number ≥ 10, may suggest typo errors (e.g. parity as 0-1-2-13). Examine those cases, correct plausible typo errors otherwise make no changes. 8.3 Among mothers who have illogical sequence of parity: 8.3.1 Expected number considerably less than the count may suggest linkage errors (e.g. parity as 0–1–2-0-1). Flag the mother as “exclusion” if expected = 1 and count ≥4, or expected ≥2 and count - expected ≥2. 8.3.2 Expected number greater than the count may suggest typo errors, especially the expected number ≥ 10. Examine those cases, correct plausible errors. 8.3.3 Make no changes for other inconsistencies (e.g. parity as 1–2-2, 0–1–2-6-4).	• 422 mothers were flagged as “exclusion”. • 161 records with plausible typo errors corrected • 36,244 mothers (4.6%) had inconsistent parity information for which no changes were made • 34,494 mothers (4.4%) might have intervening births interstates or overseas.
Consistency across different data sources			
9	Consistency in baby DOB		
	Perinatal, Hospital, ED	Validation studies reported accuracy of baby DOB in perinatal data (referred to as perinatal DOB), this variable can be used to assess the baby’s DOB and age in other linked records (referred to as patient DOB). Vice versa, when the baby DOBs across data sources differ, patient DOB can be used to verify baby DOB recorded in perinatal data. 9.1 Remove duplicates and mothers flagged as “exclusion” from perinatal data; 9.2 Combine hospital, ED and death records of the children. Remove records with invalid patient YOB (<1920 or >2014), then generate the list of patient DOBs; 9.3 Compare perinatal DOB with patient DOBs. If the perinatal DOB does not match with patient DOBs, then: 9.3.1 Identify the mothers and extract maternal hospital admission records. 9.3.2 Compare perinatal and patient DOBs with dates of maternal admission and separation. If only patient DOB matches (admission date ≤ patient DOB ≤ separation date) consider patient DOB as an alternative DOB for the baby. 9.3.3 Prior to accepting the alternative DOB, make sure that the maternal admission indicates birth delivery and the alternative DOB is not equal to the DOB of another child born to the same mother (likely linkage errors among children, except plural births), and does not create inconsistencies in pregnancy interval and parity (outlined in Steps 6 and 8). 9.4 Update the perinatal baby DOB. Merge the updated DOB into the original hospital and ED data to identify erroneous records for which correction of baby’s age or deletion of record is necessary. Correct baby’s age if the updated and patient DOBs contain the same month and year, same month and day, same day and year, or the two DOBs are less than 20 weeks apart; otherwise flag the record for “deletion”.	• Alternative DOB was created for 667 babies. The original and alternative DOBs were 1 day apart (30%), between 2 and 10 days apart (46%), and share the same day and year (16%). • Baby’s age was corrected in 41,516 hospital and ED records; • 937 hospital and ED records were flagged “deletion”.
10	Consistency between perinatal and congenital condition data		
	Perinatal, Congenital (NSW only)	10.1 Identify baby who had a linked birth defect notification; 10.2 Compare baby DOB and birthweight recorded in birth defect data versus those two variables recorded in perinatal data; 10.3 If both pieces of information differ, then identify the mother and review all pregnancy records of the mothers and the related birth defect records.	• 1 mother flagged as “exclusion” (review indicated linkage errors among her children).
11	Consistency in mother’s year of birth (YOB)		
	Perinatal, Hospital, ED, Death	11.1 Examine and define the range of mother YOBs according to perinatal data; 11.2 Examine the distribution of mother YOBs in other data sets; 11.3 Combine perinatal, hospital, ED and death data sets. Remove records with invalid mother YOBs (<1900 or >2014). For each mother: 11.3.1 Quantify how many records with an out-of-range YOB (i.e. outside the range defined at Step 11.1); 11.3.2 Quantify how many different YOBs are recorded; 11.4 Flag the mother as “exclusion” if she has ≥2 records with an out-of-range YOB or >3 different YOBs.	• YOBs in perinatal data ranged between 1941 and 1999, with no invalid values. • Hospital and ED data contain records with invalid YOBs (<1900 or >2014) and those outside the range. • 27 mothers flagged as “exclusion”;
12	Mother’s sex as male		
	Perinatal, Hospital, ED, Death	12.1 Combine mother’s perinatal, hospital, ED and death records, then remove records with invalid mother YOBs (<1900 or >2014). For each mother: 12.1.1 Quantify how many records with sex recorded as “male”; 12.1.2 Quantify how many different YOBs are record; 12.1.3 Quantify how many different months of birth are recorded; 12.2 Flag the mother as “exclusion” if she has ≥2 records with sex as male and more than one YOB and/or month of birth.	• Hospital and ED data of the mothers contain records with sex recorded as male; • 38 mothers flagged as “exclusion”
13	Mother having births after total hysterectomy procedures		
	Perinatal Hospital	13.1 Identify mothers who had hospital admissions for hysterectomy procedure(s)^j^; 13.2 Among these mothers, extract the hospital admissions during which hysterectomy procedure(s) were undertaken, and extract their most recent pregnancy record; 13.3 Compare the most recent date of delivery with date of separation following the hysterectomy procedure. Flag “exclusion” if date of separation is earlier than date of delivery.	• 51 mother flagged as “exclusion”
14	Baby DOB being later than date of discharge from hospital or ED		
	Perinatal, Hospital, ED	14.1 Combine babies’ hospital admission and ED data sets. 14.2 Compare the updated baby DOB (Step 9) with date of separation and patient DOB. Identify records where updated baby DOB is later than the date of separation. 14.3 Flag the baby as “exclusion” if the two DOBs are more than 20 weeks apart; otherwise mark the records as “deletion”. When the child is flagged as “exclusion”, further identify and flag the mother as “exclusion”.	• 10 mothers flagged as “exclusion”; • 42 hospital and ED records marked as “deletion”.
15	Date of death being earlier than episodes of health service use		
	Death Perinatal, Hospital, ED	15.1 Identify persons (mothers or babies) who have a linked death record; 15.2 Extract and combine hospital, ED and perinatal records of these persons; 15.3 Compare the person’s date of death versus date of discharge from hospital or ED and date of delivery (applicable to mothers); 15.4 Flag the person as “exclusion” if date of death is earlier than date of health service use (allowing for administrative delay of up to 3 days). If it is the case for the child, further identify and flag the mother as “exclusion”.	• 22 mothers flagged as “exclusion”.
16	Date of admission or arrival to ED being later than date of discharge		
	Hospital, ED	16.1 Identify hospital admission and ED records wherein date of admission to hospital or date of arrival to ED is later than the date of discharge. Mark these records as “deletion”.	• 34 hospital or ED records marked as “deletion”

^a^In this study, adding a variable to a data set is referred to as “merge” while adding records is referred to as “combine”

^b^It is useful to examine status of patient at discharge and date of discharge in hospital or ED data relating these deaths if dates of death differ

^c^Information useful for review: baby DOB, plurality, birth order, birthweight, gestational age, Apgar scores, discharge status, mother’s age, postcode, country of birth and hospital

^d^Perinatal data cover births that gestation ≥20 weeks or birthweight ≥400 g

^e^Birth order indicates the order each baby was born (coded as 1st, 2nd, 3rd, etc.). Birth order for singletons is 1st. Multi-fetal pregnancies generated two or more perinatal records which contain baby-specific information including order of birth while maternal information is the same

^f^In complicated plural pregnancies, it might be possible that babies born days apart, thus the gap between baby DOBs needs to be consistent with the difference in gestational age

^g^Parity as zero in the second record indicates an error, given parity defined as the number of previous pregnancies ≥20 weeks; but this errors is not always identified through the check of parity sequence (e.g. parity values as missing-0-1)

^h^The interval (calculated as in Step 6) between the first and the second record

^i^The interval between the record with missing parity and the prior record

^j^Hospital procedures were coded according to Australian Classification of Health Interventions (ACHI). See the Additional file 1 for hysterectomy procedure codes

On-screen scrutiny of relevant records was undertaken (as indicated in Table 2) when multiple entries of the same death (Step 1) or birth (Step 3) were suspected (i.e. partial duplicates), using additional information (e.g. demographic details, birthweight, Apgar scores, delivery hospital, hospital diagnoses and discharge status). Manual review of these records was time efficient because inconsistencies were found in a small number of cases.

Identified inconsistences were categorised as person-level or record-level. Person-level inconsistencies suggest likely false positive links and the persons were flagged for “exclusion” from future data analyses. Examples include a woman who conceived a second child before delivering her first child (Step 6) or had a baby after a total hysterectomy procedure (Step 13). In some cases, errors were identified for a child (e.g. date of admission later than date of death) while no inconsistencies were identified for the mother. For those cases, the mother and records for all of her children were flagged for “exclusion”.

Findings including duplicates, missing data, invalid data or likely typographical errors, and where date of admission was later than date of discharge were considered random and at record-level. Duplicates were flagged, and missing or typographical errors were corrected if plausible. Hospital separation and ED records found to contain inconsistent dates of birth, admission and discharge (Steps 9, 14 and 16) were flagged for “deletion”. Inconsistencies for which no changes were made were quantified and documented for consideration in specific analyses.

At the completion of each step, new variables were created and merged into the original data sets rather than deleting records or overwriting data values, this allowed the original data content to remain unmodified. For efficiency, decisions reached through each cleaning step were applied before undertaking the subsequent step (e.g. removal of duplicates and the use of corrected birth order to select one record per pregnancy).

#### Steps to check cross-jurisdictionally linked data

Table 3 and Fig. 2 present steps (17 to 22) to resolve discrepancies in the linkage performed by different linkage units and assess validity of apparent cross-State links. Specifically, cases where a PBS PATID matched to multiple mumPPNs were detected and sent to the AIHW linkage unit for review, through which clusters of mumPPNs (i.e. records likely to belong to the same woman) were identified (Step 17) and assessed for person-level consistency (Step 19). Step 20 examined consistency among records for women who had records in both States. Following the creation of the variable finalPPNmum (Step 21) to integrate mother’s records, consistency was checked for finalPPNmums that had multiple PATIDs (Step 22).

**Table 3:** Extract recommended PBS links and steps undertaken to check cross-jurisdictional linkage

Step Data sets		Explanation^a^	Findings
	Extract recommended PBS links		
	PBS PATID- mumPPN mapping tables	Extract the PBS links from the mapping tables where weight of the match is equal to or greater than the recommended threshold (≥29 for NSW and ≥28 for WA).	• 512,887 (50.9%) PBS links to NSW mumPPNs extracted. • 113,085 (49.9%) PBS links to WA mumPPNs extracted.
17	Detection of clusters of mumPPNs		
	PBS mapping tables	17.1 Among the extracted PBS links, identify cases where a PATID matches to two or more mumPPNs. Send these cases to AIHW data linkage unit for review and obtain advice on reliability of the matches. 17.2 It was advised that there are cases wherein all matches are correct. Consider these cases as clusters of mumPPNs 17.3 For the remaining cases, advice was given on how to select the reliable matches and reject the others.	• 9190 cases (20,254 matches) detected and reviewed. Of those, there were 3404 mumPPN clusters (including 6819 matches to 6815 mumPPNs). • 7649 PBS links were disregarded.
18	Date of pharmaceutical dispensing later than date of death		
	PBS mapping tables, PBS claims, Death	18.1 For any women who have a death record, identify the matched PATIDs (remaining at completion of Step 17); 18.2 Extract PBS claim records for those PATIDs and compare dates of pharmaceutical supply with the date of death. Disregard the PBS links if date of supply >date of death.	• 79 PBS links were disregarded.
19	Consistency in clusters of mumPPNs		
	PBS mapping tables, Perinatal, Hospital, ED, Death	For 3404 clusters (identified in Step 17): 19.1 Create the variable ClusterID and assignto all mumPPNs in each cluster. 19.2 Extract perinatal, hospital, ED and death records for these clusters. If one of the mumPPNs in the cluster has a death record or “exclusion” flag (results of Steps 1–16), assign the date of death and “exclusion” flag to the ClusterID. 19.3 Apply Steps 6, 8, 11 and 15 (outlined in Table 2) to assess consistency within each ClusterID; 19.4 Reject the cluster if it has “exclusion” flag or data inconsistencies. Further mark “exclusion” for all mumPPNs in the rejected cluster and disregard associated PBS links.	• 81 clusters were rejected, due to either - “exclusion” flag (*n* = 13); - negative pregnancy interval (*n* = 44); - different years of birth (*n* = 21); - inconsistent parity (*n* = 48); - service use after date of death (*n* = 0). • 149 mumPPNs further marked as “exclusion”; • 230 PBS links were further disregarded. • 3323 clusters were accepted.
20	Consistency in women who had records from both States		
	PBS mapping tables, Perinatal, Hospital, ED, Death	20.8 Among the remaining PBS links (at completion of Step 19), identify PATIDs which concurrently match to NSW mumPPNs and WA mumPPNs; 20.9 Create variable CrossID and assign to all mumPPNs in the pairs. 20.10 Extract perinatal, hospital, ED and death records for these CrossID. If a mumPPN in the pair has a death record or an “exclusion” flag, assign the date of death and “exclusion” flag to the CrossID; 20.11 Apply Steps 6, 8, 11 and 15 (outlined in Table 2) to assess consistency within each CrossID. 20.12 Reject the CrossID if it has “exclusion” flag or data inconsistencies. Disregard the match to mumPPN in one State and accept the match to mumPPN in the other State. The decision about which State the match to be disregarded is made based on the weight of the match (lower weight) or the State of the “exclusion” flag (e.g. exclusion flag arising from NSW data cleaning then disregard the match to NSW mumPPN).	• 2855 PATIDs concurrently match to mumPPNs in both NSW and WA; • 2645 CrossIDs were created, taking into account the network among PATIDs and mumPPNs^b^; • 659 CrossIDs were rejected, due to either - “exclusion” flag (*n* = 12); - negative pregnancy interval (*n* = 327); - different years of birth (*n* = 444) - inconsistent parity (*n* = 273); - service use after date of death (*n* = 0); • 802 PBS links were further disregarded; • 1986 CrossIDs accepted as women having births in both States.
21	Integration of mothers’ records		
	All datasets relating to mothers	Integration of records is required for mumPPN clusters and women having records in both States. 21.2 Generate a list of unique mumPPNs (as per State linkages) together with the “exclusion” flag (results of Steps 1–16 and 19). Merge in the accepted ClusterIDs and CrossIDs (Steps 19 and 20). 21.3 Create variable finalPPNmum by collapsing 3 variables according to the following hierarchy: accepted CrossID, accepted ClusterID and mumPPN; 21.4 Merge the variables finalPPNmum and “exclusion” flag into perinatal, hospital, ED, death data sets, and the mapping tables (at the completion of Step 20).	• As per State linkages, there were 783,471 mumPPNs. • Based on the variable finalPPNmum, there were 778,154 women (including those flagged as “exclusion”).
22	Consistency in finalPPNmums with multiple PATIDs and finalise mother cohort		
	PBS mapping table, PBS claims, Perinatal	22.1 Combine the NSW and WA mapping tables (at completion of Step 21), Remove PBS links relating to “exclusion” women. Among the remaining links, identify finalPPNmums that match to ≥2 PATIDs. For those women: 22.1.1 Examine the consistency of month and year of birth recorded in PBS claim records 22.1.2 Examine the consistency of parity in perinatal data (Step 8). 22.2 Flag the women as “exclusion” if month and/or year of birth is inconsistent, or sequence of parity values is illogical. Further remove related PBS links. 22.3 Extract PBS claims records for the final cohort of mothers.	• 4601 finalPPNmums with multiple PATIDs. Of those, 2763 were further flagged as “exclusion” • 7378 PBS links related to “exclusion” women were removed. • The final cohort included 774,449 women, (excluding 3705 women flagged as “exclusion”); • The final mapping table included 609,834 links, • 14,212,785 claims records were extracted.

^a^In this study, adding a variable to a data set is referred to as “merge” while adding records is referred to as “combine”

^b^For example, a NSW mumPPN matches to 2 PATIDs and these 2 PATIDs match to three different WA mumPPNs

All analyses were performed in SAS 9.3. Samples of SAS codes are provided in Additional file 1.

## Results

The checks for consistency of State-based data (Table 2) suggested false links for 703 women in NSW (0.12%) and 90 women in WA (0.05%), and flagged these women for “exclusion”. Corrections were made in 2062 perinatal records for variables including birth order (10 records), parity (1379 records), and baby date of birth (673 records) and in 41,516 hospital separation and ED records for baby’s age.

Assessing cross-jurisdictional links (Table 3), Step 19 flagged an additional 149 mumPPNs as “exclusion” and confirmed 3323 clusters of mumPPNs while Step 20 identified 1986 women who had records in both States (Step 20). Records of these mumPPNs clusters and cross-State mothers were integrated through the construction of the variable finalPPNmum (Step 21) which were used as the new person number for the mothers. The last step further identified 2763 finalPPNmums for “exclusion”, bringing the total number of women flagged for “exclusion” from future data analyses to 3705. The final cohort included 774,449 women and 1,225,341 babies born between 2003 and 2012. In this cohort, about 4.6% of women had the expected number of pregnancies greater than the number of deliveries recorded in the perinatal data, suggestive additional births elsewhere, and 4.5% had likely errors in the recording of parity. In 1838 cases, finalPPNmums were matched to two or more PBS PATIDs.

From the original mapping tables (shown in Table 1), 625,972 PBS links with weight ≥ recommended threshold were extracted and among those, 16,138 matches (2.6%) were further disregarded (Table 3). For the remaining 608,834 matches, 14,212,875 claims records were subset for the final mother cohort.

## Discussion

In this perinatal cross-jurisdictional data linkage study, we developed a series of steps to identify, and where appropriate, correct inconsistent data values. The methods were based on standard and reliable content data items [22, 23], and thus can be adopted in other perinatal research. The methods included a stepwise approach to resolving disparities in linkage performed by different linkage units and identifying women who had records in more than one State, for whom integration of records is required for analyses.

Data errors are commonly detected incidentally during statistical analyses or interpretation of results, leading to inefficient checking of data and repeating analyses [9, 11] and, potentially, lack of reproducibility of results if ad-hoc or undocumented data edits are made. We found inconsistencies that were indicative of false positive links and clusters of women’s IDs which suggest missed State-based links. These findings were fed back to the State-based data linkage units for further examination and rectification prior to future linkages, conferring benefits for other data users. Researchers play an important role in contributing to quality assurance, through systematic assessment of data consistency, given that content data have not traditionally been accessible to data linkage units under the “best practice” protocol [16–18]. The detection of the probable missed links improved data completeness, matching a further 448 perinatal records to records of maternal hospital admission for the delivery. Assessment of the consistency of the recording of parity identified women who might have additional births elsewhere (4.6%) and who had likely errors in the recording of parity (4.5%). Obstetric history is particularly important for longitudinal analyses or evaluation of interventions or exposures in the period between pregnancies.

In this study, the proportion of NSW women who were flagged for “exclusion” was lower than the false positive rate estimated by the data linkage unit in NSW (0.12% vs. 0.3%), while for WA women these proportions were similar (0.05% vs. 0.05%). This study was unable to examine the characteristics of the unlinked perinatal records, while previous studies have reported that unmatched records might hold different maternal and pregnancy characteristics compared to fully linked records [6, 7, 24]. Limitations in the data cleaning methods should also be acknowledged. Assessment of parity was less likely to detect link errors among women with fewer perinatal records, and the cut-off to flag “exclusion” due to inconsistencies in parity and mother YOB was based on a conservative decision. Given discrepancy in baby date of birth found in 667 perinatal records (0.05% of the babies), birth registrations as an additional data source would potentially helpful in assessing these discrepancies. Following the checks for clusters of mumPPNs within a PBS PATID (Step 19), an anomaly in the opposite direction (i.e. clusters of PBS PATIDs within a finalPPNmum) was present among 1838 cases (Step 22). For these women, the recording of parity, month and year of birth were consistent but no further checks using dispensing data were performed. Checking the consistency of clinical information against medicines dispensed was deemed inappropriate given that maternal morbidities recorded in the perinatal, hospital and ED data might not require a pharmacotherapy. Furthermore, our PBS data extract did not contain records for all medicines, nor did the PBS data contain records for all subsidised medicines dispensed (i.e. prior to April 2012 only subsidised medicines dispensed to social security beneficiaries were captured completely) [25]. The presence of more than one identifier in the PBS data suggests that more pharmaceutical dispensing will be attributed to these women, perhaps inappropriately, hence sensitivity analyses excluding these women should be considered.

The data cleaning process outlined in this manuscript can be summarised into stages that can be adopted in studies based on administrative health data. Moreover, majority of the specific checks undertaken in this study are generalizable to other studies. As a first step, it is important to gather necessary information to inform the development of a data cleaning plan. These include descriptions of the data collections, the variables and associated data dictionaries, the reliability of the recording of these variables as well as the procedures through which the project’s data were linked. It is advisable that the researcher examines the distribution of data (e.g. frequency, cross-tabulation), unusual patterns of the data should be discussed with the data custodians and researchers with experience working with the same data source.

It was noticed in this study that, for example, hospital records of healthy newborns were included in NSW data but were typically excluded (84%) from WA hospital admission data.

Subsequently, it is essential to draft a plan, outlining general rules about decisions to be made for identified errors, and content of specific checks (i.e. objectives and detailed algorithms). Factors to consider when creating general rules include whether there will be data sharing among analysts or use of the data for multiple research objectives, potential causes of errors (e.g. incorrect links, inconsistent patient response, inaccurate recording, typographical errors) and possible implications of decisions. Data in this project are used for several sub-studies, therefore, no deletion or overwriting of the original data value was made, instead, flag variables and corrected data values were added. Data analysts were provided with detailed documentation including noting of inconsistencies for which no changes were made so that informed decisions could be made for specific analyses. The decision regarding how to handle an error was guided by the probable cause of the error. Flags for exclusion were applied to the mother (thus, all her children) even if a linkage error was found for a child, because excluding only the problematic pregnancy record could affect analyses that investigate or control for outcomes of the prior pregnancy or health service utilisation (e.g. medication use, hospital procedures) between pregnancies. Where possible, missing, invalid and erroneous data was corrected. Flags for deletion were applied to ED or hospital records which contained inconsistencies in dates. Duplicates were flagged for removal. No changes were made for “grey” unexplainable inconsistencies.

In terms of planning for specific consistency checks, a structured approach should be used to ensure that important aspects are covered and to avoid digressing. Factors that can be used to inform which data items should be checked and the sequence of the checks include the methods of the linkage (i.e. deterministic, probabilistic), the base data set and its variables (i.e. the data sets used to derive the study population), commonalities between data sets, the coherence between different pieces of information that relate to the same event, the uniqueness of an event or expected findings, and likely consequences of unmanaged inconsistencies. It is easier to conduct the checks in the order of increasing complexity, such as commencing the checks of data items within a record, followed by examining consistencies between records of the same data set before linking records across data sets.

Our check for missing death registration record (Step 2) applicable for only NSW death data demonstrates the application of the “uniqueness” rationale that can be applied for all studies and data sources. For projects that involved cross-jurisdictionally linked data, the checks for consistency in the IDs matching (e.g. Steps 17) illustrate the effective “uniqueness” rationale to identify potential incorrect links when the study participants were represented by different sets of IDs. In studies when the IDs mapping tables are not provided by the cross-jurisdictional data linkage unit (i.e. the IDs were embedded in the data sets), researchers are advised to create the mapping tables by summarising the IDs variables to identify inconsistencies. Checking for consistency among people identified as moving between jurisdictions and the integration of IDs (Steps 19–21) are essential for all studies using cross-jurisdictional linkage of person-level unit records. A failure to identify and manage the IDs matching inconsistencies would result in a lost (if one-to-many merging) or over-collation (if many-to-many merge) of information.

During the development of algorithms, it is critical to make sure that the exclusion of study participants is not related to their health status or outcomes (i.e. the algorithms not creating selection bias). This selection bias can arise because people having multiple contacts with health services would have higher chance of inconsistencies being identified. The decision to classify the inconsistencies as incorrect links, therefore, should be based on biological and chronological plausibility, and coherence between different data items. Inconsistencies that are biologically and/or chronologically impossible (e.g. different women mapped to a single ID of the child, medications dispensed years after date of death) are indicative of incorrect linkage. When linkage errors cannot be ruled out immediately, additional information obtained from related variables or records can help to inform decisions. For example, dates of the maternal hospital separation associated with the delivery were used to verify baby DOB (Steps 6 and 9) or inconsistencies were found in more than one data items (mother’s sex and month/year of birth as in Step 12). When decisions about reasonable values or patterns are imposed, it is important to evaluate the implications of chosen cut-offs by quantifying extent of the exclusion. For instance, a conservative decision was made for inconsistencies in parity (Step 8.3.1) as a less restrictive criteria i.e. expected number of pregnancy =1 and the count of pregnancy record ≥3 (instead of ≥4) would result in an additional 156 women being flagged for exclusion (578 instead of 422 women).

## Conclusion

In conclusion, comprehensive and well-documented data consistency checks prior to commencing planned statistical analyses will improve the quality and reproducibility of perinatal research using linked administrative data. The data cleaning methods developed for the Smoking MUMS Study are recommended in other perinatal linkage studies, with appropriate modifications made based on knowledge about the data collections, validity and coherence of data items. Adoption of similar data cleaning methods across studies will assist in making comparisons across jurisdictions and countries, as well as across studies that are using ostensibly the same source datasets.

## Additional file

Additional file 1: Examples of SAS codes used for the Smoking MUMS Study data cleaning. (DOCX 69 kb)

## Abbreviations

ACT: Australian Capital Territory
APDC: Admitted Patient Data Collection
COD URF: Causes Of Death Unit Record File
DOB: Date of birth
ED: Emergency department
EDDC: Emergency Department Data Collection
HMDC: Hospital Morbidity Data Collection
MNS: Midwives Notification System
NSW: New South Wales
PATID: Project-specific Patient Identification Number (PBS linkage)
PBS: Pharmaceutical Benefits Scheme
PDC: Perinatal Data Collection
PPN: Project-specific person number (State-based linkage)
RBDM: Registry of Births, Deaths and Marriages
RoCC: Register of Congenital Conditions
WA: Western Australia
YOB: Year of birth
