# Supplementary material for: Data cleaning and management protocols for linked perinatal research data: a good practice example from the Smoking MUMS (Maternal Use of Medications and Safety) Study
Source: BMC Med Res Methodol. 2017 Jul 11;17:97. doi: 10.1186/s12874-017-0385-6 (PMC5504784; doi:10.1186/s12874-017-0385-6)
Supplement: Additional file 1: — Examples of SAS codes used for the Smoking MUMS Study data cleaning. (DOCX 69 kb) [file 12874_2017_385_MOESM1_ESM.docx]

**Additional file 1**

*** Date : October 2016;

*** Author : Dr Tran DT - University of New South Wales, Australia

*** Purpose : Examples of SAS codes used for cleaning data of the Smoking MUMS Study as reported in the manuscript titled "Tran et al. Data cleaning and management protocols for linked perinatal research data: A good practice example from the Smoking MUMS (Maternal Use of Medications and Safety) Study";

*** ID variables

mumPPN : unique ID of the mother

babyPPN : unique ID of the baby

recidPDC : unique ID of the record in the PERINATAL dataset

recidAPDC : unique ID of the record in the HOSPITAL dataset

recidEDDC : unique ID of the record in the EDDC dataset

*** Key variables in the perinatal data

bdob : Baby date of birth (DOB)

plural : Plurality (e.g. singletons, twins, etc.)

plurnum : Order of birth in each delivery (e.g. 1st, 2nd, 3rd)

pregnum : Parity (number of previous pregnancies >=20 weeks)

gestwk : Gestational age (completed weeks of gestation)

*** Key variables in other datasets

birth_date : Baby date of birth at admission or ED presentation

death_date : Date of death

admdate : Date of hospital admission

sepdate : Date of hospital separation discharge

arrdate : Date of arrival to ED

depdate : Date of departure from ED

sepmode : Mode of separation (e.g. home discharge, transfer, died, etc.)

dxP : Principal diagnose of the hospital admission

dx1 dx2 : Additional diagnoses of the hospital admission (upto 55 in NSW)

*************************************************************************

*** STEP 1: Identify duplicates

*** Outcomes: Flag "duplicates" in all datasets

*************************************************************************;

*** Flag identical duplicates in PERINATAL data;

**proc** **sort** data=PERINATAL nodupkey out=peri_nodup dupout=peri_dup;

by _______ /* list all variables, except the variable "recidPDC" */;

**run**;

**data** peri_dup;

set peri_dup (keep=recidPDC);

Duplicate=**1**; label Duplicate='Flag: duplicates';

**run**;

**proc** **sql**;

create table PERINATAL2 as select a.*, b.Duplicate from PERINATAL as a

LEFT JOIN peri_dup as b on a.recidPDC=b.recidPDC;

**quit**;

*** Flag identical duplicates in HOSPITAL data;

**proc** **sort** data=HOSPITAL_MUM nodupkey out=Hosp_nodup dupout=Hosp_dup;

by _______ /* list all variables, except the variable "recidAPDC" */;

**run**;

**data** Hosp_dup;

set Hosp_dup (keep=recidAPDC);

Duplicate=**1**; label Duplicate='Flag: duplicates';

**run**;

**proc** **sql**;

create table HOSPITAL_MUM2 as select a.*, b.Duplicate from HOSPITAL_MUM as a

LEFT JOIN Hosp_dup as b on a.recidAPDC=b.recidAPDC;

**quit**;

*** Repeat these for other datasets;

*****************************************************************************

*** STEP 3: Uniqueness of the babyPPN

*** Outcomes: Flag "duplicates" for other identified records

*** Flag exclusion "mum_excl_nonuniqbaby"

*****************************************************************************;

*** De-dup and identify babyPPN that present in 2 or more records;

**data** Baby_nonunique;

set PERINATAL2 ;

if duplicate=**1** then delete;

**run**;

**proc** **summary** data=Baby_nonunique nway;

class babyPPN;

output out = Baby_nonunique_Count (rename=(_type_=flagbaby));

**run**;

**data** Baby_nonunique_CountGE2;

set Baby_nonunique_Count ;

where _freq_ >=**2**;

keep babyPPN flagbaby ;

**run**;

**proc** **sql**;

create table Baby_nonunique2 as select * from Baby_nonunique as a

LEFT JOIN Baby_nonunique_CountGE2 as b on a.babyPPN=b.babyPPN;

**quit**;

*** Identify mothers of these non-unique babies & work with these mothers' records;

**proc** **summary** data=Baby_nonunique2 nway;

class mumPPN;

where flagbaby=**1**;

output out=Mum_nonuniqbaby (drop= _type_ _freq_) ;

**run**;

**proc** **sql**;

create table Baby_nonunique3 as select a.* from Baby_nonunique2 as a

RIGHT JOIN Mum_nonuniqbaby as b on a.mumPPN=b.mumPPN;

**quit**;

*** IF different mothers being mapped to the same baby -> Flag mother as "exclusion";

**proc** **sort** data=baby_nonunique3; by babyPPN mumPPN; **run**;

**data** Baby_nonunique4;

set Baby_nonunique3;

by babyPPN mumPPN;

lag_mum=lag(mumPPN);

if first.babyPPN then lag_mum= **.** ;

if babyPPN ne **.** and lag_mum ne **.** and mumPPN ne lag_mum then flag_diffmum=**1**;

**run**;

**proc** **sort** data=Baby_nonunique4; by babyPPN descending flag_diffmum; **run**;

**data** Baby_nonunique4;

set Baby_nonunique4;

by babyPPN descending flag_diffmum;

if first.babyPPN then diffmum=flag_diffmum; retain diffmum;

**run**;

**proc** **summary** data=Baby_nonunique4 nway;

class mumPPN;

where diffmum=**1**;

output out=mum_excl_nonuniqbaby1 (rename=(_type_=mum_excl_nonuniqbaby)
 drop=_freq_);

**run**;

*** Remove records of "exclusion" mothers before manual review;

**proc** **sql**;

create table Baby_nonunique5 as select * from Baby_nonunique4 as a

LEFT JOIN mum_excl_nonuniqbaby1 as b on a.mumPPN=b.mumPPN;

**quit**;

**data** Baby_nonunique5;

set Baby_nonunique5;

if mum_excl_nonuniqbaby=**1** then delete;

**run**;

**proc** **sort** data=baby_nonunique5; by mumPPN bdob; **run**;

*** Select perinatal variables for manual review. It is useful to review relevant information in hospital, ED or death records of the baby and/or of the mother to confirm duplicates (e.g. same babyPPN with different BDoB);

**proc** **print** data=Baby_nonunique5 (obs=**500**) noobs ;

var mumPPN babyPPN recidPDC flagbaby bdob pregnum plural plurnum
 bweight gestwk apgar1 apgar5 ;

**run**;

*** Merge "exclusion" mother (mum_excl_nonuniqbaby1) into the main "PERINATAL"

*** Execute results of the manual review;

**proc** **sql**;

create table PERINATAL3 as select * from PERINATAL2 as a

LEFT JOIN mum_excl_nonuniqbaby1 as b on a.mumPPN=b.mumPPN;

**quit**;

**data** PERINATAL3;

set PERINATAL3;

/* Make sure to include decisions (exclusion, duplicate) as per review*/

label mum_excl_nonuniqbaby='Exclusion: Nonuniqbaby';

**run**;

**proc** **datasets** lib=work nolist;

delete Baby_nonunique Baby_nonunique2 Baby_nonunique3 Baby_nonunique4 Baby_nonunique5 Baby_nonunique_count Baby_nonunique_countge2 Mum_nonuniqbaby;

**quit**;

*****************************************************************************

*** STEP 5: Birth order and pregnancy plurality

*** Outcomes: variable "plurnum_new" (as corrected birth order)

*****************************************************************************;

*** Remove duplicate perinatal records ;

**data** Birthorder;

set PERINATAL3 ;

if duplicate=**1** then delete;

keep mumPPN babyPPN recidPDC bdob plural plurnum pregnum bweight
 gestwk apgar1 apgar5 ;

**run**;

*** Select plural pregnancies;

*** Create expected sequence of birth: taking into account baby could be born in different dates;

**data** plural;

set birthorder;

if plural >**1** then output;

**run**;

**proc** **sort** data=plural; by mumPPN bdob plurnum; **run**;

**data** plural;

set plural;

by mumPPN bdob plurnum;

* Calcualte difference in dates of birth between 2 consecutive records;

lagdate=lag(bdob); format lagdate ddmmyy10.;

if first.mumPPN then lagdate=**.**;

if lagdate ne **.** then do ; diffday=bdob-lagdate; end;

* Calcualte difference in gestation (weeks) between 2 consecutive records;

lagga=lag(gestwk);

if first.mumPPN then lagga=**.**;

if lagga ne **.** and gestwk ne **.** then do diffga=gestwk-lagga; end;

* Create sequence number: start from 1 and increase by 1, given below
 conditions;

if first.bdob then seqorder=**0**; retain seqorder; seqorder+**1**;

if seqorder>**0** and **0**< abs(diffga***7**-diffday)<**7** then seqorder=seqorder+**1**;

if seqorder ne plurnum then flag_check=**1**;

**run**;

*** Identify mothers and all of their records for review;

**proc** **summary** data=plural nway;

class mumPPN;

where flag_check=**1**;

output out=mum_review;

**run**;

**proc** **sql**;

create table birthorder_review as select a.* from birthorder as a

RIGHT JOIN mum_review as b on a.mumPPN=b.mumPPN;

**quit**;

**proc** **sort** data=birthorder_review; by mumPPN bdob plurnum; **run**;

**proc** **print** data=birthorder_review (obs=**150**) noobs; **run**;

*** Create the variable plurnum_new in the main PERINATAL dataset

This new variable will be used in the subsequent analyses;

**data** PERINATAL4;

set PERINATAL3;

/* Excute the decisions according to the review */

label plurnum_new='Birth order corrected';

**run**;

**proc** **datasets** lib=work nolist;

delete Birthorder plural mum_review birthorder_review;

**quit**;

***************************************************************************

*** STEP 6: Birth interval

*** Outcomes: Flag exclusion "mum_excl_neginter"

***************************************************************************;

*** De-dup and select records where birth order=1st;

**data** Interval;

set PERINATAL4 ;

if duplicate=**1** then delete;

If plurnum_new=**1** then output; * This variable is created in Step 5;

keep mumPPN babyPPN bdob gestwk ;

**run**;

*** Create variable "Interval"

Flag the record where interval less than zero;

**proc** **sort** data=Interval; by mumPPN bdob; **run**;

**data** Interval;

set Interval;

by mumPPN bdob;

format condate priordate ddmmyy10.;

condate= bdob-gestwk***7** +**14**;

priordate=lag(BDOB); if first.mumPPN then priordate=**.**;

period=condate-priordate - **7**;

if **.** < period < **0** then flag_negative=**1**;

**run**;

*** IF mothers had any birth interval<0 -> Mark "exclusion";

**proc** **summary** data=Interval nway;

class mumPPN;

where flag_negative=**1**;

output out= mum_excl_neginter (rename=(_type_=mum_excl_neginter) drop=_freq_);

**run**;

*** Merge these "exclusion" mothers in the main PERINATAL dataset;

**proc** **sql**;

create table PERINATAL5 as select * from PERINATAL4 as a

LEFT JOIN mum_excl_neginter as b on a.mumPPN=b.mumPPN;

**quit**;

**data** PERINATAL5 ;

set PERINATAL5;

label mum_excl_neginter='Exclusion: Negative Interval';

**run**;

**proc** **datasets** lib=work nolist; delete Interval ; **quit**;

*************************************************************************

*** STEP 7: Replacing missing value of parity

*** Outcomes: new variable "parity" (as corrected parity value)

*************************************************************************;

*** Remove duplicate records ;

**data** Missingparity;

set PERINATAL5 ;

if duplicate=**1** then delete;

condate=bdob-gestwk***7**+**14**; format condate ddmmyy10. ;

if pregnum=**.** then flagmissing=**1**;

keep mumPPN babyPPN recidPDC bdob gestwk pregnum
 plural plurnum_new condate flagmissing ;

**run**;

*** Identify mums with missing parity and work with these mothers;

**Proc** **summary** data=Missingparity nway;

class mumPPN;

where flagmissing=**1**;

output out=mumwithmissing;

**run**;

**proc** **sql**;

create table Missingparity2 as select a.* from Missingparity as a

RIGHT JOIN mumwithmissing as b on a.mumPPN=b.mumPPN;

**quit**;

*** Quantity missing parity in plural births. Given there are not many cases, choose to review and make changes;

**proc** **freq** data=Missingparity2; table plural; where flagmissing=**1**; **run**;

**proc** **summary** data=Missingparity2 nway;

class mumPPN;

where flagmissing=**1** and plural >**1**;

output out =mumwithmissing_plural ;

**run**;

**proc** **sql**;

create table plural_review as select a.* from Missingparity2 as a

RIGHT JOIN mumwithmissing_plural as b on a.mumPPN=b.mumPPN;

**quit**;

**proc** **sort** data=plural_review; by mumPPN bdob; **run**;

**proc** **print** data=plural_review noobs; **run**;

*** Replace missing parity in plural births after review;

**data** Missingparity3;

set Missingparity2;

/* Make changes according to the review

Make sure to include these changes in the main PERINATAL */

**run**;

*** Now, select only one record per pregnancy;

**data** Missingparity4;

set Missingparity3;

where plurnum_new=**1**;

drop plurnum_new;

**run**;

*** Create variable "nrec" to indicate the number of perinatal records of each mother;

**proc** **sort** data=Missingparity4; by mumPPN BDob; **run**;

**data** Missingparity4;

set Missingparity4;

by mumPPN BDOB;

if first.mumPPN then seq=**0**; seq+**1**;

**run**;

**proc** **sort** data=Missingparity4; by mumPPN descending seq; **run**;

**data** Missingparity4;

set Missingparity4;

by mumPPN descending seq;

if first.mumPPN then nrec=seq; retain nrec;

**run**;

*** Create variable "cntmissing" to indicate the number of records with missing parity;

**proc** **sort** data=Missingparity4; by mumPPN seq; **run**;

**data** Missingparity4;

set Missingparity4;

by mumPPN seq;

if first.mumPPN then missing_seq=**.**; retain missing_seq;

missing_seq =sum(missing_seq, flagmissing);

**run**;

**proc** **sort** data=Missingparity4; by mumPPN descending missing_seq; **run**;

**data** Missingparity4;

set Missingparity4;

by mumPPN descending missing_seq;

if first.mumPPN then cntmissing=missing_seq; retain cntmissing;

**run**;

*** Create variable "messyorder" to indicate that the mother has illogical order of the parity (non-missing);

**proc** **sort** data=Missingparity4; by mumPPN descending flagmissing bdob; **run**;

**data** Missingparity4;

set Missingparity4;

by mumPPN descending flagmissing bdob ;

priorparity_nonmiss=lag(pregnum);

if first.mumPPN then priorparity_nonmiss=**.**;

if pregnum ne **.** and pregnum <=priorparity_nonmiss then flag_messyorder=**1**;

**run**;

**proc** **sort** data=Missingparity4; by mumPPN descending flag_messyorder; **run**;

**data** Missingparity4;

set Missingparity4;

by mumPPN descending flag_messyorder;

if first.mumPPN then messyorder=flag_messyorder; retain messyorder;

**run**;

*** Create variable "zero_secondpreg" to indicate that the mother has parity=0 in the second record (i.e. an error);

**data** Missingparity4;

set Missingparity4;

if seq=**2** and pregnum=**0** then flag_zero_secondpreg=**1**;

**run**;

**proc** **sort** data=Missingparity4; by mumPPN descending flag_zero_secondpreg; **run**;

**data** Missingparity4;

set Missingparity4;

by mumPPN descending flag_zero_secondpreg;

if first.mumPPN then zero_secondpreg =flag_zero_secondpreg;

retain zero_secondpreg;

**run**;

*** NO CHANGES for mothers who either had only 1 perinatal record, >=2 records with missing parity, parity=0 in the second, or illogical sequence -> Remove these mothers;

**data** Missingparity5;

set Missingparity4;

if nrec=**1** or cntmissing>=**2** or messyorder=**1** or zero_secondpreg=**1** then delete;

drop missing_seq cntmissing priorparity_nonmiss flag_messyorder messyorder
 flag_zero_secondpreg zero_secondpreg ;

**run**;

*** Among the remaining mothers, load information from a record to the 2 adjacent records (prior and next);

**proc** **sort** data=Missingparity5; by mumPPN seq; **run**;

**data** Missingparity5;

set Missingparity5;

by mumPPN seq;

* Lag parity to the next record;

priorparity=lag(pregnum); if first.mumPPN then priorparity=**.**;

* Lag BDOB to the next record;

priorBDoB=lag(BDOB); if first.mumPPN then priorBDOB=**.**;

format priorBDoB ddmmyy10.;

* Calculate the interval (from the prior delivery) ;

if condate ne **.** then priorperiod=(condate-priorBDoB)/**7**;

**run**;

**proc** **sort** data=Missingparity5; by mumPPN descending seq; **run**;

**data** Missingparity5;

set Missingparity5;

by mumPPN descending seq;

* Lag parity to the prior record;

nextparity=lag(pregnum); if first.mumPPN then nextparity=**.**;

* Lag conception date (not BDOB) to the prior record;

nextcondate=lag(condate); if first.mumPPN then nextcondate=**.**;

format nextcondate ddmmyy10.;

* Calculate the interval (to the conception of the next pregnancy);

if nextcondate ne **.** then nextperiod=(nextcondate-BDoB)/**7**;

**run**;

**proc** **sort** data=Missingparity5; by mumPPN seq; **run**;

*** Replace missing parity given following conditions;

**data** Missingparity5;

set Missingparity5;

by mumPPN seq;

YOB=year(BDoB);

if last.mumPPN then last=**1**;

* Replace missing parity in the first record;

if pregnum=**.** and seq=**1** then do;

if nextparity in (**1**) then replace_parity=nextparity-**1**;

else if nextparity>**1** & nextperiod <=**40** then replace_parity=nextparity-**1**;

end;

* Replace missing parity in the last record;

else if pregnum=**.** and last=**1** then do;

replace_parity=priorparity+**1**;end;

* Replace missing parity in record other than the first and last;

else if pregnum =**.** and last ne **1** and seq ne **1** then do;

if nextparity-priorparity =**2**

then replace_parity=priorparity+**1**;

else if nextparity-priorparity >**2** & priorperiod<=**40**

then replace_parity=priorparity+**1**;

end;

**run**;

*** Merge "replace_parity" to the main PERINATAL dataset

Make sure to include above results of manual review among plural births

Create variable "parity" as corrected value of of parity;

**data** replace_parity;

set Missingparity5;

if replace_parity ne **.** then output;

keep mumPPN recidPDC replace_parity;

**run**;

**proc** **sql**;

create table PERINATAL6 as select a.*, b.replace_parity from PERINATAL5 as a

LEFT JOIN replace_parity as b on a.recidPDC=b.recidPDC;

**quit**;

**data** PERINATAL6;

set PERINATAL6;

/* Make sure to include results of plural births manual review */

** Update the parity for all mothers;

parity=pregnum;

if pregnum eq **.** and replace_parity ne **.** then parity=replace_parity;

Label parity='Corrected parity'; drop replace_parity;

**run**;

**proc** **datasets** lib=work nolist;

delete Missingparity mumwithmissing Missingparity2 mumwithmissing_plural

plural_review Missingparity3 Missingparity4 Missingparity5 ;

**quit**;

***********************************************************************

*** STEP 8: Consistency of parity

*** Based on the corrected "parity" (created in Step 7)

*** Outcomes: Flag exclusion "mum_excl_parity"

**********************************************************************;

*** De-dup and select one record per pregnancy (plurnum_new=1);

**data** checkparity;

set PERINATAL6 ;

if duplicate=**1** then delete;

if plurnum_new =**1** then output;

keep mumPPN babyPPN recidPDC bdob gestwk parity ;

**run**;

*** Flag missing parity & "count" the number of perinatal records of each mother;

**proc** **sort** data=checkparity; by mumPPN bdob; **run**;

**data** checkparity;

set checkparity;

by mumPPN bdob;

if parity=**.** then flagmissing=**1**;

if first.mumPPN then seq=**0**; seq+**1**;

**run**;

**proc** **sort** data= checkparity; by mumPPN descending seq; **run**;

**data** checkparity;

set checkparity;

by mumPPN descending seq;

if first.mumPPN then count=seq; retain count;

**run**;

*** Create variable "maxparity" & "minparity" to indicate the maximum /minimum value of the parity;

**proc** **sort** data=checkparity; by mumPPN flagmissing descending parity; **run**;

**data** checkparity;

set checkparity;

by mumPPN flagmissing descending parity;

if first.mumPPN then maxparity=parity; retain maxparity;

**run**;

**proc** **sort** data= checkparity; by mumPPN flagmissing parity; **run**;

**data** checkparity;

set checkparity;

by mumPPN flagmissing parity;

if first.mumPPN then minparity=parity; retain minparity;

**run**;

*** Create variable "expect" to indicate the expected number of pregnancy;

*** Create variable "messyorder" to indicate mother has illogical sequence of parity values (non-missing, sorted according to bdob);

**proc** **sort** data= checkparity; by mumPPN flagmissing bdob; **run**;

**data** checkparity;

set checkparity;

by mumPPN flagmissing bdob;

expect=maxparity-minparity+**1**;

priorp=lag(parity);

if first.mumPPN then priorp=**.**;

if parity ne **.** and priorp ne **.** and priorp>=parity then flag_messyorder=**1**;

**run**;

**proc** **sort** data= checkparity; by mumPPN descending flag_messyorder; **run**;

**data** checkparity;

set checkparity;

by mumPPN descending flag_messyorder;

if first.mumPPN then messyorder=flag_messyorder; retain messyorder;

**run**;

**proc** **sort** data= checkparity; by mumPPN seq; **run**;

*** IF mothers have highly inconsistent parity ->Flag "exclusion";

**data** mum_excl_parity;

set checkparity;

If messyorder= **1** then do;

if expect=**1** and count>=**4** then mum_excl_parity=**1**;

else if expect>**1** and count-expect >=**2** then mum_excl_parity=**1**;

end;

if mum_excl_parity=**1** and seq=**1** then output;

keep mumPPN mum_excl_parity ;

**run**;

*** Merge these "exclusion" mothers in the main PERINATAL dataset;

**proc** **sql**;

create table PERINATAL7 as select a.*, b.mum_excl_parity from PERINATAL6 as a

LEFT JOIN mum_excl_parity as b on a.mumPPN=b.mumPPN;

**quit**;

**data** PERINATAL7;

set PERINATAL7;

Label mum_excl_parity ='Exclusion: Inconsistent parity';

**run**;

**proc** **datasets** lib=work nolist; delete checkparity checkparity2 ; **quit**;

***********************************************************************************

*** STEP 9: Consistency in Baby DOB

*** Outcomes: new variable "bdob_new" as corrected baby DOB

*** Flag "deletion_babyDOB" in hospital/ED data of the baby

**********************************************************************************;

*** De-dup and extract baby DOB from perinatal records,

*** Make sure to remove mothers who were flagged "exclusion" due to non-unique babyPPN (Step 3);

**data** Peri_bdob;

set PERINATAL7;

If duplicate=**1** then delete;

If Mum_excl_nonuniqbaby=**1** then delete;

if babyPPN =**.** then delete;

keep mumPPN babyPPN bdob ;

**run**;

*** De-dup and extract babies' hospital admission, ED and death records

Combine the datasets and remove records where babyYOB is invalid ;

**data** Deathbaby_bdob;

set Death_baby2 ;

if duplicate=**1** then delete;

keep babyPPN birth_date;

**run**;

**data** Hospbaby_bdob ;

set HOSPITAL_BABY2 ;

if duplicate=**1** then delete;

keep babyPPN birth_date;

**run**;

**data** EDbaby_bdob;

set EDDC_baby2 ;

if duplicate=**1** then delete;

keep babyPPN birth_date;

**run**;

**data** Combine_bdob;

set Deathbaby_bdob Hospbaby_bdob EDbaby_bdob;

**run**;

**data** Combine_bdob2;

set combine_bdob;

bYOB=year(birth_date);

if bYOB <**1920** or bYOB >**2014** then delete; * Invalid babyYoB;

**run**;

*** Compile the list of babyDOB in the patient's data;

**proc** **summary** data=Combine_bdob2 nway;

class babyPPN birth_date ;

output out=Summary_birthdate (drop=_type_ rename =(_freq_=ptfreq));

**run**;

*** Merge perinatal DOB with the summarised patient data;

**proc** **sql**;

create table compare_bdob as select * from Peri_bdob as a

INNER JOIN summary_birthdate as b on a.babyPPN=b.babyPPN;

**quit**;

**data** compare_bdob;

set compare_bdob;

if bdob =birth_date then flag_match=**1**;

**run**;

**proc** **sort** data=compare_bdob; by babyPPN descending flag_match ; **run**;

**data** compare_bdob;

set compare_bdob;

by babyPPN descending flag_match;

if first.babyPPN then match= flag_match; retain match ;

**run**;

*** IF dates of birth matching-> No changes to be made-> Remove the matching babies. IF dates are not matching -> Extract all the values of date of birth

(in this study, a baby could have 4 baby DOB values (1 perinatal + 3 patient);

**data** compare_bdob2;

set compare_bdob;

if match =**1** then delete;

drop flag_match match;

**run**;

**proc** **sort** data=compare_bdob2; by babyPPN descending ptfreq ; **run**;

**data** compare_bdob2;

set compare_bdob2;

by babyPPN descending ptfreq ;

if first.babyPPN then seq=**0**; seq+**1**;

**run**;

**proc** **freq** data=compare_bdob2; table seq/missing; **run**;

**data** date1

date2 (rename=(birth_date=birth_date2))

date3 (rename=(birth_date=birth_date3));

set compare_bdob2;

if seq=**1** then output date1 ;

else if seq=**2** then output date2 ;

else if seq=**3** then output date3 ;

drop seq;

**run**;

**proc** **sql**;

create table compare_bdob3 as select a.*, b.birth_date2, c.birth_date3 from

date1 as a LEFT JOIN date2 as b on a.babyPPN=b.babyPPN

LEFT JOIN date3 as c on a.babyPPN=c.babyPPN;

**quit**;

*** Extract mothers' hospital records, compare baby DOB values with the mother's dates of admission and separation;

**data** Hospitalmum_dates;

set HOSPITAL_MUM2 ;

if duplicate=**1** then delete;

keep mumPPN admdate sepdate dxP dx1 dx2;

**run**;

**proc** **sql**;

create table compare_bdob_hospmum as select * from Hospitalmum_dates as a

RIGHT JOIN compare_bdob3 as b on a.mumPPN=b.mumPPN;

**quit**;

**data** compare_bdob_hospmum2;

set compare_bdob_hospmum;

if admdate <=bdob<=sepdate then perimatch=**1**;

if admdate <=birth_date <=sepdate then patientmatch_cat=**1**;

else if admdate <=birth_date2 <=sepdate then patientmatch_cat=**2**;

else if admdate <=birth_date3 <=sepdate then patientmatch_cat=**3**;

if perimatch=**.** & patientmatch_cat =**.** then delete;

if admdate=**.** then delete;

**run**;

*** IF perinatal baby DOB matches with maternal admission-> No changes -> Remove these babies. IF only patient baby DOB matches -> Choose as an alternative baby DOB;

**proc** **sort** data=compare_bdob_hospmum2; by babyPPN descending perimatch; **run**;

**data** compare_bdob_hospmum2;

set compare_bdob_hospmum2;

by babyPPN descending perimatch;

if first.babyPPN then nochange=perimatch; retain nochange;

**run**;

**data** compare_bdob_hospmum3;

set compare_bdob_hospmum2;

if nochange=**1** then delete;

format bdob_2nd ddmmyy10.;

if patientmatch_cat=**1** then bdob_2nd=birth_date;

else if patientmatch_cat=**2** then bdob_2nd=birth_date2;

else if patientmatch_cat=**3** then bdob_2nd=birth_date3;

drop perimatch nochange;

**run**;

*** Examine maternal hospital diagnoses;

**proc** **tabulate** data=compare_bdob_hospmum3 missing;

class dxP dx1 dx2 patientmatch_cat ;

table dxP * dx1* dx2 , patientmatch_cat N;

**run**;

*** Get the unique record for each baby

*** Examine the difference between the original and corrected baby DOB;

**proc** **sort** data=compare_bdob_hospmum3 nodupkey out=bdob_2nd dupout=dup ; by babyPPN bdob_2nd ; **run**;

**data** bdob_2nd ;

set bdob_2nd ;

keep babyPPN bdob bdob_2nd mumPPN;

**run**;

**data** bdob_2nd_check ;

set bdob_2nd ;

dif=abs(bdob- bdob_2nd);

if year(bdob_2nd)=year(bdob) & month(bdob_2nd)=month(bdob)

then cat='Diff Day';

else if year(bdob_2nd)=year(bdob) & day(bdob_2nd)=day(bdob)

then cat='Diff Mth';

else if month(bdob_2nd)=month(bdob) & day(bdob_2nd)=day(bdob)

then cat='Diff Yr';

**run**;

**proc** **freq** data=bdob_2nd_check;

table dif * cat/norow nocol nopercent missing ;

**run**;

**proc** **tabulate** data=bdob_2nd_check missing ;

class dif cat;

table cat;

table dif , cat/ nocellmerge;

**run**;

*** Check whether this alternative baby DOB is equal to DoB of other babies born to the same mother;

*** Other checks include whether alternative baby DOB cause negative birth interval or parity inconsistency;

**proc** **sql**;

create table Peri_bdob2nd_check as select a.*, b.babyPPN2, b.bdob_2nd

from Peri_bdob as a RIGHT JOIN

bdob_2nd (rename=(babyPPN=babyPPN2)) as b on a.mumPPN=b.mumPPN;

**quit**;

**data** Peri_bdob2nd_check;

set Peri_bdob2nd_check;

if babyPPN ne babyPPN2 and bdob=bdob_2nd then check1=**1**;

**run**;

**proc** **freq** data=Peri_bdob2nd_check; table check1 ; **run**;

**proc** **print** data=Peri_bdob2nd_check; var mumPPN; where check1=**1**; **run**;

**proc** **print** data=Peri_bdob2nd_check noobs; where mumPPN in ( ); **run**;

** Merge alternative baby DOB to the main PERINATAL dataset and update baby DOB;

**proc** **sql**;

create table PERINATAL8 as select a.*, b.bdob_2nd from PERINATAL7 as a

LEFT JOIN bdob_2nd as b on a.babyPPN=b.babyPPN;

**quit**;

**data** PERINATAL8;

set PERINATAL8;

bdob_new=bdob;

if bdob_2nd ne **.** then bdob_new=bdob_2nd;

Label bdob_new ='Updated babyDoB'; format bdob_new ddmmyy10.;

**run**;

*** Merge the updated baby DOB to the main HOSPITAL_BABY and EDDC_BABY

to flag record for "deletion" due to discrepancy in baby dates of birth;

**data** updatedBDob;

set PERINATAL8;

If duplicate=**1** then delete;

If Mum_excl_nonuniqbaby=**1** then delete;

if babyPPN =**.** then delete;

keep babyPPN bdob_new;

**run**;

**proc** **sql**;

create table HOSPITAL_BABY3 as select * from HOSPITAL_BABY2 as a

LEFT JOIN updatedBDob as b on a.babyPPN=b.babyPPN;

**quit**;

**data** HOSPITAL_BABY3;

set HOSPITAL_BABY3;

if (**0**<=abs(bdob_new-birth_date)<=**140**) OR

(year(bdob_new)=year(birth_date) & month(bdob_new)=month(birth_date)) OR

(year(bdob_new)=year(birth_date) & day(bdob_new)=day(birth_date)) OR

(day(bdob_new)=day(birth_date) & month(bdob_new)=month(birth_date))

then match=**1**;

if match ne **1** & (bdob_new ne birth_date) then deletion_babyDOB=**1**;

label deletion_babyDOB='Deletion: Discrepant babyDOB';

drop bdob_new match;

**run**;

**proc** **sql**;

create table EDDC_BABY3 as select * from EDDC_BABY2 as a

LEFT JOIN updatedBDob as b on a.babyPPN=b.babyPPN;

**quit**;

**data** EDDC_BABY3;

set EDDC_BABY3;

if (**0**<=abs(bdob_new-birth_date)<=**140**) OR

(year(bdob_new)=year(birth_date) & month(bdob_new)=month(birth_date)) OR

(year(bdob_new)=year(birth_date) & day(bdob_new)=day(birth_date)) OR

(day(bdob_new)=day(birth_date) & month(bdob_new)=month(birth_date))

then match=**1**;

if match ne **1** & (bdob_new ne birth_date) then deletion_babyDOB=**1**;

label deletion_babyDOB='Deletion: Discrepant babyDOB';

drop bdob_new match;

**run**;

**proc** **datasets** lib=work nolist;

delete Peri_bdob Deathbaby_bdob Hospbaby_bdob EDbaby_bdob Combine_bdob Combine_bdob2

Summary_birthdate compare_bdob compare_bdob2 compare_bdob3 Hospitalmum_dates

compare_bdob_hospmum compare_bdob_hospmum2 compare_bdob_hospmum3 Dup

Peri_bdob2nd_check updatedBDob ;

**quit**;

**proc** **freq** data=HOSPITAL_BABY3; table deletion_babyDOB; **run**;

**proc** **freq** data=EDDC_BABY3; table deletion_babyDOB; **run**;

********************************************************************

*** STEP 10: Mother YOB

*** Outcomes: Flag exclusion "mum_excl_YOB"

********************************************************************;

*** De-dup and combine mother's Perinatal, hospital admission, ED & death records;

**data** Peri_mumYOB;

set PERINATAL8;

if duplicate=**1** then delete;

source=**1**;

keep mumPPN mumYOB mumMOB source ;

**run**;

**data** Death_mumYOB;

set DEATH_MUM2;

if duplicate=**1** then delete;

source=**2**;

keep mumPPN mumYOB mumMOB source;

**run**;

**data** Hospital_mumYOB;

set HOSPITAL_MUM2 ;

if duplicate=**1** then delete;

source=**3**;

keep mumPPN mumYOB mumMOB source;

**run**;

**data** ED_mumYOB;

set EDDC_MUM2 ;

if duplicate=**1** then delete;

source=**4**;

keep mumPPN mumYOB mumMOB source;

**run**;

**data** Combine_mumYOB;

set Peri_mumYOB Hospital_mumYOB ED_mumYOB Death_mumYOB;

**run**;

*** Examine distribution of mumYOB;

**proc** **freq** data=Combine_mumYOB;

table mumYOB * source/norow nocol nopercent missing;

**run**;

*** Remove records where mumYOB missing or invalid,

Flag out-of-range mumYOB;

**data** Combine_mumYOB2;

set Combine_mumYOB;

if mumYOB <**1900** or mumYOB>**2014** then delete;

if **1900** <=mumYOB<**1941** or **1999**< mumYOB <=**2014** then outrange=**1**;

**run**;

*** IF mother had >1 records with out-of-range mumYOB -> Flag "exclusion";

**proc** **summary** data=Combine_mumYOB2 nway;

class mumPPN ;

where outrange=**1**;

output out=cnt_outrange;

**run**;

**data** mum_excl_YOB1 ;

set cnt_outrange (rename=(_type_=mum_excl_YOB1));

where _freq_>**1** ;

keep mumPPN mum_excl_YOB1;

**run**;

*** IF mother had >3 different values of mumYOB -> Flag "exclusion";

**proc** **summary** data=Combine_mumYOB2 nway;

class mumPPN mumYOB;

output out=Summary_mumYOB;

**run**;

**proc** **summary** data=Summary_mumYOB nway;

class mumPPN;

output out=CntmumYOB;

**run**;

**data** mum_excl_YOB2 ;

set CntmumYOB (rename=(_type_=mum_excl_YOB2));

where _freq_>**3** ;

keep mumPPN mum_excl_YOB2;

**run**;

*** Collate the 2 lists of 'exclusion' and generate the list of unique mumPPN;

**data** mum_excl_YOB;

set mum_excl_YOB1 mum_excl_YOB2 ;

**run**;

**proc** **sort** data=mum_excl_YOB; by mumPPN; **run**;

**data** mum_excl_YOB;

set mum_excl_YOB;

by mumPPN;

mum_excl_YOB=**1**;

if first.mumPPN then output;

keep mumPPN mum_excl_YOB;

**run**;

*** Merge these "Exclusion" mothers in the main PERINATAL dataset;

**proc** **sql**;

create table PERINATAL9 as select * from PERINATAL8 as a

LEFT JOIN mum_excl_YOB as b on a.mumPPN=b.mumPPN;

**quit**;

**data** PERINATAL9;

set PERINATAL9;

Label mum_excl_YOB ='Exclusion: Inconsistent mumYOB';

**run**;

**proc** **datasets** lib=work nolist;

delete Peri_mumYOB Death_mumYOB Hospital_mumYOB ED_mumYOB Combine_mumYOB Combine_mumYOB2 cnt_outrange Summary_mumYOB CntmumYOB mum_excl_YOB1 mum_excl_YOB2;

**quit**;

**************************************************************************

*** STEP 12: Mother's sex as Male in hospital and ED data

*** Outcomes: Flag exclusion "mum_excl_sex"

**************************************************************************;

*** De-dup and combine mother's Perinatal, hospital admission, ED & death records;

**data** Peri_sex;

set PERINATAL9;

if duplicate=**1** then delete;

source=**1**;

keep mumPPN mumYOB mumMOB source;

**run**;

**data** Death_sex;

set DEATH_MUM2 ;

if duplicate=**1** then delete;

source=**2**;

keep mumPPN mumYOB mumMOB source;

**run**;

**data** Hospital_sex;

set HOSPITAL_MUM2 ;

if duplicate=**1** then delete;

source=**3**;

keep mumPPN mumYOB mumMOB source sex dxP dx1 dx2;

**run**;

**data** ED_sex;

set EDDC_MUM2 ;

if duplicate=**1** then delete;

source=**4**;

keep mumPPN mumYOB mumMOB source sex ;

**run**;

**data** Combine_sex;

set Peri_sex Death_sex Hospital_sex ED_sex ;

**run**;

*** Remove records where YOB is invalid and create mother date of birth;

**data** Combine_sex2;

set Combine_sex;

if mumYOB <**1900** or mumYOB>**2014** then delete;

mumDOB=mdy(mumMOB, **1**, mumYOB); format mumDOB monyy7.;

**run**;

**proc** **freq** data= Combine_sex2; table sex * source/ norow nocol nopercent missing; **run**;

*** Count number of Male records per mother;

**proc** **summary** data=Combine_sex2 nway;

class mumPPN ;

where sex=**1**;

output out=cntmum_sex (rename=(_freq_=cntsex ) drop=_type_);

**run**;

**proc** **freq** data=cntmum_sex; table cntsex/missing; **run**;

*** Count number of mumDOB values per mother;

**proc** **summary** data=Combine_sex2 nway;

class mumPPN mumDOB;

output out=Summary_mumDOB;

**run**;

**proc** **summary** data=Summary_mumDOB nway;

class mumPPN;

output out=CntmumDOB (rename=(_freq_=cntmumDOB ) drop=_type_);

**run**;

**proc** **freq** data=CntmumDOB; table cntmumDOB /missing; **run**;

*** IF mothers have >1 "Male" records and >1 DOB values -> Flag "exclusion";

**Proc** **sql**;

create table Summary_sexMYOB as select * from CntmumDOB as a

RIGHT JOIN cntmum_sex as b on a.mumPPN=b.mumPPN;

**quit**;

**data** Mum_excl_sex;

set Summary_sexMYOB;

mum_excl_sex=**1**;

if CntmumDOB>**1** and cntsex>**1** then output;

keep mumPPN mum_excl_sex;

**run**;

*** Merge these "Exclusion" mothers in the main PERINATAL dataset;

**proc** **sql**;

create table PERINATAL10 as select * from PERINATAL9 as a

LEFT JOIN Mum_excl_sex as b on a.mumPPN=b.mumPPN;

**quit**;

**data** PERINATAL10;

set PERINATAL10;

Label Mum_excl_sex ='Exclusion: Sex';

**run**;

**proc** **datasets** lib=work nolist;

delete Peri_sex Death_sex Hospital_sex ED_sex Combine_sex Combine_sex2 cntmum_sex Summary_mumDOB CntmumDOB Summary_sexMYOB ;

**quit**;

**************************************************************************

*** STEP 13: Have birth after a total hysterectomy procedure

*** Outcomes: Flag exclusion "mum_excl_TAH"

***************************************************************************;

*** Identify hysterectomy admissions, search in the principal & 15 additional procedure fields;

**data** Mum_TAH;

set HOSPITAL_MUM2 ;

if duplicate=**1** then delete;

ARRAY hysterectomy {*} procedure_codeP procedure_code1 - procedure_code15;

do j=**1** to **16**;

if hysterectomy {j} in:

('35653-00', '35653-01', '35653-02', '35653-03', '35653-04', '35661-00', '35664-00', '35667-00', '35670-00', '90448-00', '90448-01', '90448-02',

'35657-00', '35664-01', '35667-01', '35673-00', '35673-01', '35673-02',

'35750-00', '35753-00', '35753-01', '35753-02', '35756-00', '35756-01', '35756-02', '35756-03', '90450-00', '90450-01', '90450-02') then flag_hysterectomy=**1**; END; drop j;

if flag_hysterectomy=**1** then output;

keep mumPPN admdate sepdate flag_hysterectomy;

**run**;

*** For each mother, select the most recent birth record;

**data** peri_mostrecent;

set PERINATAL10;

if duplicate=**1** then delete;

where plurnum_new=**1**;

**run**;

**proc** **sort** data=peri_mostrecent; by mumPPN bdob_new; **run**;

**data** peri_mostrecent;

set peri_mostrecent;

by mumPPN bdob_new;

if last.mumPPN then output;

keep mumPPN bdob_new;

**run**;

*** IF procedure was before the most recent delivery -> Flag "exclusion";

**proc** **sql**;

create table lastbirth_proc as select * from Mum_TAH as a

LEFT JOIN peri_mostrecent as b on a.mumPPN=b.mumPPN;

**quit**;

**data** lastbirth_proc;

set lastbirth_proc;

if sepdate < BdoB_new then flag_excl_TAH=**1**;

**run**;

**proc** **summary** data=lastbirth_proc nway;

class mumPPN;

where flag_excl_TAH=**1**;

output out= mum_excl_TAH (rename=(_type_=mum_excl_TAH) drop=_freq_);

**run**;

*** Merge these "exclusion" in the main PERINATAL dataset;

**proc** **sql**;

create table PERINATAL11 as select * from PERINATAL10 as a

LEFT JOIN mum_excl_TAH as b on a.mumPPN=b.mumPPN;

**quit**;

**data** PERINATAL11;

set PERINATAL11;

Label mum_excl_TAH ='Exclusion: Hysterectomy';

**run**;

**proc** **datasets** lib=work nolist;

delete peri_mostrecent lastbirth_proc APDC_TAH HMDC_TAH Mum_TAH;

**quit**;

***********************************************************************************

*** STEP 14: Child admission or ED presentation before born

*** Based on the corrected baby DOB

*** Outcomes: Flag exclusion "mum_excl_admb4born"

*** Flag deletion "deletion_admb4born" in baby's hospital/ED records

**********************************************************************************;

*** De-dup perinatal, remove "exclusion" mothers (due to non-unique babyPPN, Step 3);

**data** Peri_admb4born;

set PERINATAL11;

If duplicate=**1** then delete;

If Mum_excl_nonuniqbaby=**1** then delete;

if babyPPN eq **.** then delete ;

keep mumPPN babyPPN bdob_new;

**run**;

*** De-dup, combine baby hospital and ED datasets, remove records with invalid DOB;

**data** Hospbaby_admB4born;

set HOSPITAL_BABY3 (rename=(admdate=startdate sepdate=enddate));

If duplicate=**1** then delete;

source=**3**;

keep babyPPN startdate enddate source birth_date recidAPDC;

**run**;

**data** EDbaby_admB4born;

set EDDC_BABY3 (rename=(arrdate=startdate depdate=enddate));

If duplicate=**1** then delete;

source=**4**;

keep babyPPN startdate enddate source birth_date recidEDDC;

**run**;

**data** HospED_admB4born;

set Hospbaby_admB4born EDbaby_admB4born;

**run**;

**data** HospED_admB4born;

set HospED_admB4born;

if year(birth_date) <**1920** or year(birth_date)>**2012** then delete;

**run**;

*** Compare perinatal baby DOB vs date of separation and patient DOB

IF perinatal DOB later than date of separation & the two DOBs > 20wks apart

-> Exclude the baby -> Identify and flag the mother "exclusion"

OTHERWISE -> Flag the hospital or ED as "deletion";

**proc** **sql**;

create table HospED_admB4born2 as select * from HospED_admB4born as a

INNER JOIN Peri_admB4born as b on a.babyPPN=b.babyPPN;

**quit**;

**data** HospED_admB4born3;

set HospED_admB4born2;

if (bdob_new > enddate >**.**) and abs(bdob_new - birth_date)>=**140** then flag_exclbaby=**1**;

**run**;

**proc** **sort** data=HospED_admB4born3; by babyPPN descending flag_exclbaby; **run**;

**data** HospED_admB4born3;

set HospED_admB4born3;

by babyPPN descending flag_exclbaby;

if first.babyPPN then exclusion_baby=flag_exclbaby; retain exclusion_baby;

**run**;

**data** HospED_admB4born3;

set HospED_admB4born3;

if exclusion_baby eq **.** and (bdob_new > enddate >**.**) then deletion=**1**;

**run**;

*** Identify mother for 'exclusion" flag;

**proc** **summary** data=HospED_admB4born3 nway;

class mumPPN ;

where exclusion_baby=**1**;

output out=mum_excl_admb4born

(rename=(_type_=mum_excl_admb4born) drop=_freq_ );

**run**;

*** Merge these "Exclusion" mothers into the MAIN perinatal record;

**proc** **sql**;

create table PERINATAL12 as select * from PERINATAL11 as a

LEFT JOIN mum_excl_admb4born as b on a.mumPPN=b.mumPPN;

**quit**;

**data** PERINATAL12;

set PERINATAL12;

Label mum_excl_admb4born ='Exclusion: baby born later than admission';

**run**;

*** Compile the lists of the record for "deletion"

Merge these "Deletion" records into the MAIN baby's hospital and ED data sets;

**data** deletion_Hospbaby;

set HospED_admB4born3;

if deletion=**1** and source=**3** then output;

keep babyPPN deletion recidAPDC;

**run**;

**proc** **sql**;

create table HOSPITAL_BABY4 as select a.*, b.deletion from HOSPITAL_BABY3 as a

LEFT JOIN deletion_Hospbaby as b on a.recidAPDC=b.recidAPDC;

**quit**;

**data** HOSPITAL_BABY4;

set HOSPITAL_BABY4 (rename=(deletion=deletion_admb4born));

label deletion_admb4born='Deletion: Born later than admission';

**run**;

**data** deletion_EDbaby;

set HospED_admB4born3;

if deletion=**1** and source=**4** then output;

keep babyPPN deletion recidEDDC;

**run**;

**proc** **sql**;

create table EDDC_BABY4 as select a.*, b.deletion from EDDC_BABY3 as a

LEFT JOIN deletion_EDbaby as b on a.recidEDDC=b.recidEDDC;

**quit**;

**data** EDDC_BABY4;

set EDDC_BABY4 (rename=(deletion=deletion_admb4born));

label deletion_admb4born='Deletion: Born later than admission';

**run**;

**proc** **datasets** lib=work nolist;

delete Peri_admb4born Hospbaby_admB4born EDbaby_admB4born HospED_admB4born

HospED_admB4born2 HospED_admB4born3 deletion_Hospbaby deletion_EDbaby ;

**quit**;

***********************************************************************************

*** STEP 15: Date of service use later than date of death (gap<=3 days allowed)

*** Outcomes: Flag exclusion "mum_excl_resurrect"

***********************************************************************************;

*** Dedup perinatal & remove "exclusion" mothers (due to nonunique babyPPN, Step 3);

**data** Peri_resurrect;

set PERINATAL12;

If duplicate=**1** then delete;

If Mum_excl_nonuniqbaby=**1** then delete;

if babyPPN =**.** then delete;

keep mumPPN babyPPN bdob_new ;

**run**;

*** Combine date of death of mothers and babies

Check whether baby was born after date of death: NO cases (codes not provided);

**data** Death_resurrect_baby;

set DEATH_BABY2;

If duplicate=**1** then delete;

PPN=babyPPN;

person=**2**;

keep PPN babyPPN death_date person;

**run**;

**data** Death_resurrect_mum;

set DEATH_MUM2;

If duplicate=**1** then delete;

PPN=mumPPN;

person=**1**;

keep PPN mumPPN death_date person;

**run**;

**data** Death_resurrect;

set Death_resurrect_mum Death_resurrect_baby;

**run**;

*** Combined mother's datasets (perinatal+ Hospital+ED)

Identify hospital and ED records that indicate a death;

**data** Perimum_resurrect;

set Peri_resurrect;

source=**1**;

person=**1**;

keep mumPPN bdob_new source person;

**run**;

**data** Hospmum_resurrect;

set HOSPITAL_MUM2 (rename=(admdate=startdate sepdate=enddate));

If duplicate=**1** then delete;

if sepmode in ('6', '7') then sepmode_died=**1**; * Check dictionary for sepmode;

source=**3**;

person=**1**;

keep mumPPN startdate enddate source person sepmode_died;

**run**;

**data** EDmum_resurrect;

set EDDC_MUM2 (rename=(arrdate=startdate depdate=enddate));

If duplicate=**1** then delete;

if sepmode in ('3', '8') then sepmode_died=**1**; * Check dictionary for sepmode;

source=**4**;

person=**1**;

keep mumPPN startdate enddate source person sepmode_died ;

**run**;

*** Combine baby datasets (ED and hospital)

Identify hospital and ED records that indicate a death;

**data** Hospbaby_resurrect;

set HOSPITAL_BABY4 (rename=(admdate=startdate sepdate=enddate));

If duplicate=**1** then delete;

If deletion_admb4born =**1** then delete;

source=**3**;

person=**2**;

if sepmode in ('6', '7') then sepmode_died=**1**; * Check dictionary for sepmode;

keep babyPPN startdate enddate source person sepmode_died;

**run**;

**data** EDbaby_resurrect;

set EDDC_BABY4 (rename=(arrdate=startdate depdate=enddate));

If duplicate=**1** then delete;

If deletion_admb4born =**1** then delete;

if sepmode in ('3', '8') then sepmode_died=**1**; * Check dictionary for sepmode;

source=**4**;

person=**2**;

keep babyPPN startdate enddate source person sepmode_died ;

**run**;

*** Combine datasets of the mothers and datasets of the babies

Create a common variable PPN for IDs of both mothers and babies

If Perinatal records, set the enddate as date of delivery;

**data** Combine_resurrect;

set perimum_resurrect Hospmum_resurrect EDmum_resurrect Hospbaby_resurrect
 EDbaby_resurrect;

**run**;

**data** Combine_resurrect;

set Combine_resurrect;

PPN=sum(babyPPN, mumPPN);

if source = **1** then enddate=bdob_new;

**run**;

*** Merge date of death into the combined dataset. IF gap>3 -> Flag person as "resurrect". IF the case for baby, identify and flag the mother "exclusion";

**proc** **sql**;

create table Combine_resurrect2 as select a.*, b.death_date from Combine_resurrect as a INNER JOIN Death_resurrect as b on a.PPN=b.PPN;

**quit**;

**data** Combine_resurrect2;

set Combine_resurrect2;

if enddate - death_date >**3** then flag_afterdeath=**1**;

**run**;

**proc** **summary** data=Combine_resurrect2 nway;

class PPN person ;

where flag_afterdeath=**1**;

output out=afterdeath (drop=_freq_ _type_);

**run**;

**data** afterdeath;

set afterdeath;

resurrect=**1**;

**run**;

**proc** **sql**;

create table excl_resurrect as select a.mumPPN, a.babyPPN, b.resurrect_mum

from Peri_resurrect as a LEFT JOIN afterdeath (rename=(resurrect=resurrect_mum)) as b on a.mumPPN=b.PPN;

**quit**;

**proc** **sql**;

create table excl_resurrect2 as select a.*, b.resurrect_baby

from excl_resurrect as a LEFT JOIN afterdeath (rename=(resurrect=resurrect_baby)) as b on a.babyPPN=b.PPN;

**quit**;

**data** excl_resurrect3;

set excl_resurrect2;

if resurrect_mum =**1** or resurrect_baby=**1** then exlc_mum_resurrect=**1**;

if exlc_mum_resurrect=**.** then delete;

**run**;

**proc** **summary** data=excl_resurrect3 nway;

class mumPPN;

where exlc_mum_resurrect=**1**;

output out=mum_excl_resurrect (rename=(_type_=mum_excl_resurrect)
 drop=_freq_);

**run**;

*** Merge these "Exclusion" mothers into the MAIN perinatal record;

**proc** **sql**;

create table PERINATAL13 as select * from PERINATAL12 as a

LEFT JOIN mum_excl_resurrect as b on a.mumPPN=b.mumPPN;

**quit**;

**data** PERINATAL13;

set PERINATAL13;

Label mum_excl_resurrect='Exclusion: service use after death';

**run**;

**proc** **datasets** lib=work nolist;

delete Peri_resurrect Perimum_resurrect Death_resurrect_baby Death_resurrect_mum Death_resurrect AFterdeath

Combine_resurrect Combine_resurrect2 EDbaby_resurrect EDmum_resurrect excl_resurrect excl_resurrect2 excl_resurrect3

Hospmum_resurrect ;

**quit**;

***********************************************************************************

*** STEP 16: Date of admission/ED arrival > date of separation/ED departure

*** Outcomes: Flag deletion "deletion_dates"

***********************************************************************************;

**data** HOSPITAL_MUM_FINAL;

set HOSPITAL_MUM2;

IF **.**< sepdate < admdate then deletion_dates=**1**;

label deletion_dates='Deletion: Start date later than End date';

**run**;

**data** HOSPITAL_BABY_FINAL;

set HOSPITAL_BABY4;

IF **.**< sepdate < admdate then deletion_dates=**1**;

label deletion_dates='Deletion: Start date later than End date';

**run**;

**data** EDDC_MUM_FINAL;

set EDDC_MUM2;

IF **.**< depdate < arrdate then deletion_dates=**1**;

label deletion_dates='Deletion: Start date later than End date';

**run**;

**data** EDDC_BABY_FINAL;

set EDDC_BABY4;

IF **.**< depdate < arrdate then deletion_dates=**1**;

label deletion_dates='Deletion: Start date later than End date';

**run**;

**proc** **freq** data=HOSPITAL_MUM_FINAL; table deletion_dates; **run**;

**proc** **freq** data=HOSPITAL_BABY_FINAL; table deletion_dates; **run**;

**proc** **freq** data=EDDC_MUM_FINAL; table deletion_dates; **run**;

**proc** **freq** data=EDDC_BABY_FINAL; table deletion_dates; **run**;

*************************************************************************

*** QUANTIFY NUMBER OF "EXCLUSION" MOTHERS

*************************************************************************;

*** Combine various exclusion flags into 1 variable in perinatal data;

**data** PERINATAL_FINAL;

SET PERINATAL13;

IF mum_excl_nonuniqbaby =**1** OR

mum_excl_neginter =**1** OR

mum_excl_parity =**1** OR

mum_excl_YOB =**1** OR

Mum_excl_sex =**1** OR

mum_excl_TAH =**1** OR

mum_excl_admb4born =**1** OR

mum_excl_resurrect =**1** THEN mum_exclusion=**1**;

**run**;

**proc** **summary** data=PERINATAL_FINAL nway;

class mumPPN;

where mum_exclusion=**1**;

output out=cnt_mum_exclusion ;

**run**;
